# Sandwich‐Structured Implants to Obstruct Multipath Energy Supply and Trigger Self‐Enhanced Hypoxia‐Initiated Chemotherapy Against Postsurgical Tumor Recurrence and Metastasis

**DOI:** 10.1002/advs.202300899

**Published:** 2023-05-08

**Authors:** Youqiang Fang, Xing Luo, Yanteng Xu, Zheng Liu, Rachel L. Mintz, Haiyang Yu, Xuan Yu, Kai Li, Enguo Ju, Haixia Wang, Zhaohui Tang, Yu Tao, Mingqiang Li

**Affiliations:** ^1^ Laboratory of Biomaterials and Translational Medicine Center for Nanomedicine, Department of Urology The Third Affiliated Hospital, Sun Yat‐sen University Guangzhou 510630 P. R. China; ^2^ Department of Biomedical Engineering Washington University in St. Louis St. Louis MO 63110 USA; ^3^ Key Laboratory of Polymer Ecomaterials Changchun Institute of Applied Chemistry Chinese Academy of Sciences Changchun 130022 P. R. China; ^4^ Department of Ultrasound The Third Affiliated Hospital Sun Yat‐sen University Guangzhou 510630 P. R. China; ^5^ Guangdong Provincial Key Laboratory of Liver Disease Research Guangzhou 510630 P. R. China

**Keywords:** 3D printing, electrospinning, hypoxia‐triggered chemotherapy, in situ delivery, starvation therapy

## Abstract

As a currently common strategy to treat cancer, surgical resection may cause tumor recurrence and metastasis due to residual postoperative tumors. Herein, an implantable sandwich‐structured dual‐drug depot is developed to trigger a self‐intensified starvation therapy and hypoxia‐induced chemotherapy sequentially. The two outer layers are 3D‐printed using a calcium‐crosslinked mixture ink containing soy protein isolate, polyvinyl alcohol, sodium alginate, and combretastatin A4 phosphate (CA4P). The inner layer is one patch of poly (lactic‐*co*‐glycolic acid)‐based electrospun fibers loaded with tirapazamine (TPZ). The preferentially released CA4P destroys the preexisting blood vessels and prevents neovascularization, which obstructs the external energy supply to cancer cells but aggravates hypoxic condition. The subsequently released TPZ is bioreduced to cytotoxic benzotriazinyl under hypoxia, further damaging DNA, generating reactive oxygen species, disrupting mitochondria, and downregulating hypoxia‐inducible factor 1*α*, vascular endothelial growth factor, and matrix metalloproteinase 9. Together these processes induce apoptosis, block the intracellular energy supply, counteract the disadvantage of CA4P in favoring intratumor angiogenesis, and suppress tumor metastasis. The in vivo and in vitro results and the transcriptome analysis demonstrate that the postsurgical adjuvant treatment with the dual‐drug‐loaded sandwich‐like implants efficiently inhibits tumor recurrence and metastasis, showing great potential for clinical translation.

## Graphical abstract

A dual‐drug‐loaded sandwich‐structured implant is developed to inhibit postsurgical tumor recurrence and metastasis. Combretastatin A4 phosphate (CA4P), preferentially released from 3D‐printed outer layers, obstructs extracellular energy supply, and aggravates hypoxia. Tirapazamine, subsequently released from the electrospun inner layer, is bioreduced under hypoxia, induces apoptosis, blocks intracellular energy supply, counteracts the drawback of CA4P in favoring angiogenesis, and prevents tumor metastasis.

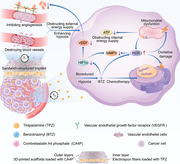

## 1 Introduction

Cancer is a growing global health threat owing to the significant morbidity and mortality in recent decades.^[^ ^1^ ^]^ The pathogeneses of cancers and their therapies have been studied extensively in both laboratory and clinical settings. Presently, the clinically effective approaches for cancer treatment mainly involve locoregional (surgery and radiation therapy) and systemic therapies. The most common treatment method, surgical resection, usually results in a high risk of tumor recurrence and metastasis, which is easily induced by the residual cancer cells.^[^ ^2^ ^]^ Radiotherapy often causes severe side effects, resulting in unavoidable pain.^[^ ^3^ ^]^ In addition, conventional chemotherapy still poses many challenges, including toxicity in benign tissues or organs and restricted drug distribution in targeted tumor tissues.^[^ ^3^, ^4^ ^]^ Although many emerging and developing cancer treatments have been reported, such as immunotherapy and gene therapy, their ambiguous therapeutic effects, latent adverse effects, high therapy costs, and low patient acceptance need further investigation and clarification, limiting their clinical translatability.^[^ ^5^ ^]^ Therefore, the development of novel therapeutic strategies that can dramatically enhance the antitumor efficacy is still highly required.

The combination treatment including tumor excision and postsurgical in situ implantation is a viable strategy, which is not only cost‐effective but also predicted to have high efficacy, low side effects, and great promise for clinical translation.^[^ ^6^ ^]^ The remaining tumor tissue after resection not only can induce tumor recurrence but also can facilitate the cancer cell migration and invasion through the bloodstream. Therefore, further killing the residual cancer cells and blocking the blood vessels at the tumor site by in situ implantations of drugs is an ideal adjuvant therapy after tumor resection, which is expected to substantially improve the anticancer efficacy. CA4P is a typical water‐soluble vascular blocking agent that can irreversibly cut off the intratumor preexisting blood vessels and suppress neovascularization during tumor growth, thus effectively obstructing the oxygen and nutrition supplied to tumors.^[^ ^7^ ^]^ As a promising starvation therapy drug, CA4P has successfully exhibited its anticancer effect in Phase II clinical trials.^[^ ^8^ ^]^ However, on the one hand, the blood vessel disruption can dramatically increase intratumor hypoxia and subsequently upregulate the intracellular hypoxia‐inducible factor 1*α* (HIF1*α*), which can enhance the expression and secretion of vascular endothelial growth factor (VEGF), further stimulating revascularization and promoting tumor metastasis.^[^ ^6^, ^9^ ^]^ On the other hand, the single obstruction of the external energy supply may not achieve efficient starvation, due to the physiological complexity and the adaptive resistibility of tumors.^[^ ^10^ ^]^ Thus, it is essential to find a cooperative chemotherapeutic agent that works in conjunction with CA4P to induce cytotoxicity in response to the elevated hypoxia and neutralize the negative conditions created by the upregulated HIF1*α*‐VEGF pathway.

Hypoxia‐activated prodrugs (HAPs) display selectively higher cytotoxicity to the tumor tissues in a hypoxic environment and lower cytotoxicity to the normal tissues under normoxic or hyperoxic conditions.^[^ ^11^ ^]^ TPZ, a representative HAP, exhibits up to 200‐fold higher cytotoxicity in the hypoxic tumor microenvironment (TME) than in a normoxic environment.^[^ ^12^ ^]^ Within the cancer cells under hypoxia, TPZ can be bioreduced to transient radical intermediates (•TPZ), accompanied by the production of reactive oxygen species (ROS) (i.e., superoxide anions and hydroxyl radicals), and then reduced to a cytotoxic product benzotriazinyl (BTZ), which can interact with and damage DNA, finally inducing apoptosis.^[^ ^12^, ^13^ ^]^ Specifically, the selective cytotoxicity of TPZ not only can take full advantage of the hypoxia reinforced by CA4P but also can tackle the ineluctable disadvantage of single treatment with CA4P. TPZ can efficiently reduce HIF1*α* and VEGF levels in cancer cells by inhibiting the intracellular HIF1*α* synthesis in the hypoxic TME.^[^ ^14^ ^]^ Besides, the TPZ‐generated free radicals and ROS can lead to mitochondrial dysfunction, which not only can severely impair the internal energy supply but also can continue to produce ROS.^[^ ^15^ ^]^ The downregulation of HIF1*α* and overaccumulation of ROS may lessen the intracellular or intratumor abundance of matrix metalloproteinases (MMPs), crucial factors favoring tumor invasion and metastasis.^[^ ^6^, ^9^ ^]^ Hence, the combination therapy with TPZ and CA4P is expected to be a promising therapeutic approach toward cancer treatment.^[^ ^16^ ^]^ Furthermore, there is currently no facile approach to prepare an implantable system that integrates CA4P and TPZ with controllable release after single dosing, which can achieve a more convenient local administration with a lower systemic side effect and sequential and long‐lasting drug actions with a higher anticancer efficacy after tumor resection, compared to the drug administration by multiple injections as free or nanoparticulate forms.

**Scheme 1:**
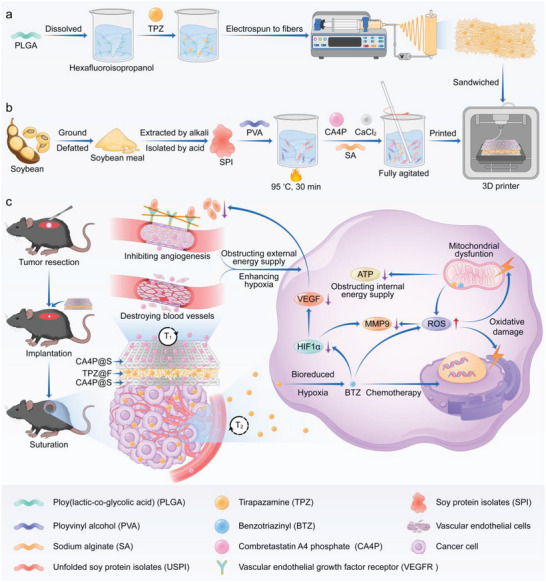
Schematic illustration of preparation and inhibition of postsurgical tumor recurrence and metastasis for sandwich‐structured composites loaded with CA4P and TPZ. a) Fabrication of PLGA‐based electrospun fibers containing TPZ. b) Preparation of SPI, SPI‐contained 3D‐printed scaffolds loaded with CA4P, and dual‐drug‐loaded sandwich‐structured composites. c) Tumor resection, composite implantation, and controlled drug release and tumor treatment mechanisms for the CA4P and TPZ in sandwich‐structured composites.

Herein, a sandwich‐structured implant consisting of two outer layers of 3D‐printed natural soy protein isolate (SPI)‐polyvinyl alcohol (PVA)‐sodium alginate (SA)‐based scaffolds loaded with CA4P and one inner layer of electrospun poly (lactic‐*co*‐glycolic acid) (PLGA)‐based fibers loaded with TPZ was proposed and fabricated to completely kill the residual cancer cells after surgical resection, thus inhibiting tumor recurrence and metastasis, and finally achieving the satisfactory therapeutic efficacy (**Scheme** **1**). The CA4P preferentially released from exterior layers blocked the external energy supply to cancer cells by destroying the well‐established blood vessels and constraining the neovascularization in tumors. Then, the TPZ released from the inner layer was bioreduced to BTZ under hypoxia promoted by CA4P. The internalization and bioreduction of TPZ induced apoptosis through damaging the nuclear DNA, generated cytotoxic free radicals and ROS, disturbed mitochondria to obstruct the internal energy supply to cancer cells, and downregulated HIF1*α*, VEGF, and MMP9 to impede angiogenesis and cancer cell migration and invasion. Consequently, the tumor excision and the customized implantable sandwich‐like depots capable of sequentially releasing CA4P and TPZ cooperatively held promise for effective tumor therapy.

## 2 Results and Discussion

### 2.1 Construction and Characterization of Electrospun Fibers, 3D‐Printed Scaffolds, and Sandwich‐Structured Composites

**Figure 1:**
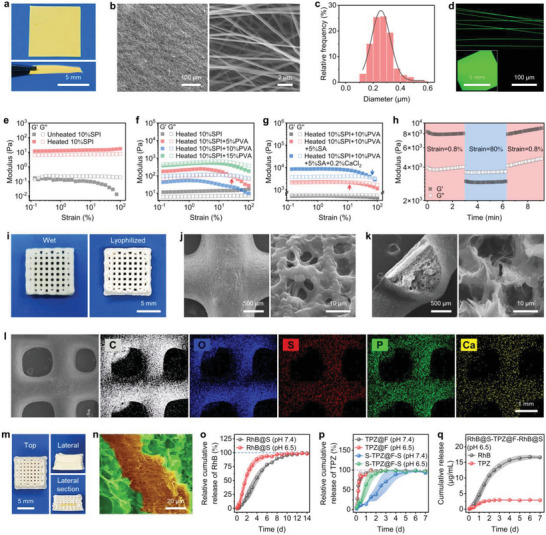
Characterizations and properties of electrospun fibers, 3D‐printed scaffolds, and sandwich‐structured composites. a) Digital macrographs, b) SEM images, c) statistical diameter distribution plot, and d) fluorescence micrographs of TPZ@F. Oscillatory strain‐dependent rheological behaviors (*G*', elastic modulus; *G*", viscous modulus) of e) unheated and heated SPI, f) heated mixtures of SPI and PVA, and g) heated mixtures of SPI and PVA with or without SA or CaCl_2_. h) Rheological thixotropy of the used 3D‐printing ink composed of a heated mixture of SPI and PVA, SA, and CaCl_2_ under an oscillation cycle model with varied strain. i) Digital macrographs, j) exterior, and k) interior microstructures reflected by the SEM images, and l) element (C, O, S, P, and Ca) distribution images based on the energy‐dispersive spectroscope (EDS) mapping of CA4P@S. m) Digital macrographs and n) pseudocolor SEM images of CA4P@S‐TPZ@F‐CA4P@S (CA4P@S, green; TPZ@F, red). o) Profiles of relative cumulative release from scaffolds for CA4P simulated by RhB with similar solubility. p) Relative cumulative release profiles of TPZ from TPZ‐loaded electrospun fibers (TPZ@F) or TPZ@F sandwiched between blank scaffolds (S‐TPZ@F‐S). q) Absolute cumulative release profiles of RhB or TPZ from the dual‐drug‐loaded sandwich‐like composites (RhB@S‐TPZ@F‐RhB@S).

The impalpable sandwich‐like composite was constructed using one patch of electrospun fibers sandwiched between two 3D‐printed scaffolds. As the interior layer, TPZ‐loaded electrospun fibers (TPZ@F) were fabricated with a mixed hexafluoroisopropanol (HFIP) solution containing PLGA and TPZ. After the complete volatilization of HFIP, fibers were cut into small patches for future use. As shown in **Figure** **1**a, a square PLGA‐based fiber patch with a side length of about 7 mm appeared yellow due to the TPZ loading. Throughout one patch, the fibers were arranged randomly and densely, with a relatively uniform diameter of ≈260.0 nm (Figure 1b,c). Through scanning the ultraviolet–visible spectrum of TPZ, we found a prominent peak absorbance at the wavelength of about 470 nm. Thus, we observed the TPZ‐loaded fibers and patches using a fluorescence microscope. As expected, both the TPZ@F and TPZ@F‐constituted patches displayed noticeable green fluorescence under the excitation of around 488 nm (Figure 1d), which also confirmed the successful loading of TPZ.

Before the fabrication of exterior layers composed of 3D‐printed scaffolds, SPI, as the critical component of printing ink, was isolated from the defatted soy powder using a combined method containing alkali extraction and acid precipitation (Scheme 1).^[^ ^17^ ^]^ The purity and composition of the obtained SPI were confirmed using sodium dodecyl sulfate‐polyacrylamide gel electrophoresis (SDS‐PAGE, Figure S1, Supporting Information) and Fourier transform infrared (FTIR) spectrum (Figure S2, Supporting Information). Then, the appropriate formula for SPI‐included printing ink was determined based on the typical rheological analysis. As presented in Figure 1e, the native SPI solution (10%, w/v) exhibited shear‐thinning rheological behaviors dominated by viscosity. The 10% heated SPI (HSPI) solution had stable and elasticity‐dominated rheological characteristics, showing that HSPI was more suitable for 3D printing. This perhaps resulted from the intramolecular or intermolecular crosslinking of SPI in a thermal environment, revealed by the SPI aggregate band in the image of nonreducing SDS‐PAGE (Figure S1b, Supporting Information). However, the viscoelasticity of HSPI solution was still too weak to maintain the initial shapes of the structures after extrusion. Thus, before the thermal treatment, we introduced a biocompatible synthetic polymer PVA, which hardly chemically reacted with SPI at normal (25 °C) or high temperatures (Figure S1, Supporting Information). With the addition of PVA, the physically crosslinked polymer networks made both the elastic modulus (*G*') and the viscous modulus (*G*") of mixtures indeed increase. Still, the bulk rheological behaviors gradually became unstable (Figure 1f). For the mixture containing 5% w/v PVA, the *G*' and *G*" profiles intersected when the oscillatory strain reached 31.6%, indicating that the viscosity became preponderated, which was undesirable. With the increasing concentrations (10% and 15%) of PVA, *G*" became higher than *G*' throughout the strain sweep process, demonstrating that the mixtures were no longer applicable to 3D printing. Considering that the high viscosity of the mixture with 15% PVA might disfavor further operation, we proceeded with the heated mixture composed of 10% SPI and 10% PVA. In response to the sequential addition of SA or CaCl_2_, the *G*' progressively exceeded the *G*", and the intersection of their profiles appeared at a higher strain (75.0%) for the mixture containing both SA and CaCl_2_ (Figure 1g). This mixture also presented the intended shear thixotropy, indicated by the excellent viscoelastic restorability under varied strains (Figure 1h). Consequently, the optimal 3D‐printing ink contained a heated mixture of 10% SPI and 10% PVA, 5% w/v SA, and 0.2% w/v CaCl_2_. Following the determination of the ink formula, the CA4P‐contained scaffolds (CA4P@S) made up of crisscrossed sticks were printed in a cubic shape with a top‐view side length of about 8.0 mm (Figure 1i). The lyophilization hardly affected the basic shapes of the printed scaffolds, further verifying the stability and machinability of the printing ink. The microstructure of the lyophilized scaffolds is shown in the scanning electron microscope (SEM) images (Figure 1j,k). The smooth exterior was dotted with irregular pores. The coarse interior was filled with closely packed holes formed by the gel network in the printing ink, which promoted the total interface area and provided more release sites for CA4P.^[^ ^18^ ^]^ The element distribution graphs based on SEM are presented in Figure 1l. In addition to the basic elements C and O, the evenly distributed elements S, P, and Ca confirmed the presence of SPI, CA4P, and CaCl_2_ in CA4P@S, respectively. Moreover, the FTIR also suggested that the fabricated scaffolds contained SPI, PVA, and SA (Figure S2, Supporting Information).

After the determination of the preparation formulas and parameters for the electrospun fibers and 3D‐printed scaffolds, the drug‐loaded sandwich‐structured composites were fabricated via sandwiching a patch of TPZ@F between two pieces of CA4P@S. The macrographs of lyophilized composites viewed from diverse angles are presented in Figure 1m. It was visible from the photograph and the SEM image of the lateral section that the TPZ@F patch was successfully sandwiched between scaffolds (Figure 1m,n). Before investigating the drug release properties of constructed scaffolds and electrospun fibers, we first explored their degradability in different environments. The SEM images in Figure S3a (Supporting Information) and the weight losses after incubation for 12 d (Figure S3b, Supporting Information) showed that the scaffolds degraded more easily at pH 6.5 than at pH 7.4. At pH 6.5, the subacid TME was simulated, and the proteases accelerated the degradation of protein‐contained scaffolds. The digital graphs in Figure S3c (Supporting Information) illustrated that the fastest degradation of PLGA‐based fiber patches also occurred at pH 6.5 in the presence of lipase, which mimicked the TME. The relatively higher degradability in TME allowed a sustained release for the drugs loaded in the scaffold‐fibers‐scaffold composites. Since CA4P was difficult to detect, Rhodamine B (RhB, peak absorbance at around 554 nm) with a similar solubility was substituted for monitoring its release in vitro. As shown in Figure 1o, RhB was almost entirely released from the scaffolds (RhB@S) after oscillation and incubation for 10 d in the phosphate‐buffered saline solution (PBS) at pH 7.4 or 6.5. The release rate of RhB at pH 7.4 was lower than that at pH 6.5, at which the subacid TME was stimulated. According to an earlier study, CA4P could be released more readily from carriers under subacid conditions.^[^ ^19^ ^]^ As illustrated in Figure 1p, TPZ in the fibers was cumulatively released by nearly 100.0% on day 3. The release rate was reduced via sandwiching the fiber patch with scaffolds, which was in accordance with our previous study.^[^ ^6c^ ^]^ By sandwiching, the influence of pH on the release rate of TPZ also became noticeable, reflected by the slower release at pH 7.4. Although the relative release rate of TPZ was faster than that of CA4P in the same environments (Figure 1o,p), the absolute cumulative release of TPZ was always smaller than the release of CA4P. The released TPZ also saturated faster than CA4P during the 7‐day release period at pH 6.5 (Figure 1q). Therefore, the CA4P in the exterior layers of sandwich‐like implants would be released first and result in cytotoxicity before the TPZ in the interior layer, which was similar to the drug release property of sandwich‐like composites previously demonstrated by our group.^[^ ^6c^ ^]^

### 2.2 In Vitro Vascular Endothelial Cells Proliferation Inhibition by CA4P@S

**Figure 2:**
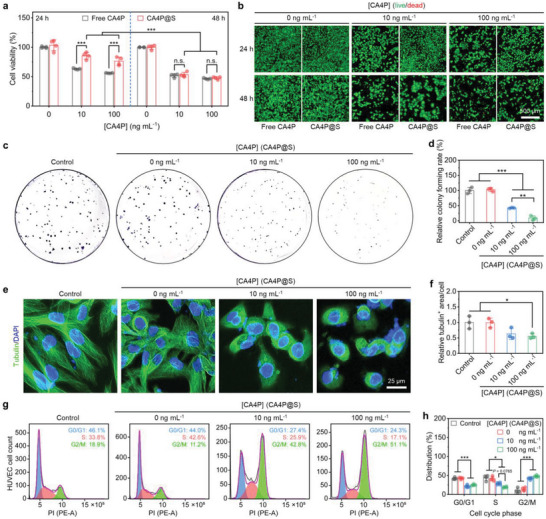
In vitro inhibitory effect of CA4P@S on the proliferation of vascular ECs. a) Cell viability and b) calcein‐AM/PI (live/dead)‐stained fluorescence images of HUVECs coincubated with free CA4P or CA4P@S for different durations. c) Digital photographs and d) relative forming rates based on the counted numbers of HUVEC colonies dyed with crystal violet after treatment with CA4P@S for 7 days. e) CLSM images and f) 2D‐viewed relative fluorescence area per cell of HUVEC microtubules stained with tubulin‐tracker green after coincubation with CA4P@S for 12 h. g) Flow cytometry analysis and h) distribution quantification for the cell cycle phase of HUVECs treated with CA4P@S for 12 h. Control group, HUVECs without the coincubation with free CA4P or CA4P@S. Data are presented as mean ± standard deviation (SD; *n* = 4 in panels a and h; *n* = 3 in panels (d) and (f). Statistical significance was evaluated by two‐way ANOVA in panels (a) and (h), and by one‐way ANOVA in panel (d) and (f). *P < 0.05, **P < 0.01, ***P < 0.001, and n.s., no significance.

To confirm the antiproliferation effect of CA4P on vascular endothelial cells (VECs), we performed cell counting kit 8 (CCK8) assays and cell live/dead staining analysis on the immortalized human umbilical VECs (HUVECs). The CCK8 results showed (**Figure** **2**a) that the HUVEC proliferation was inhibited after the coincubation with free CA4P or CA4P@S, and the inhibitions were increasingly improved with the prolonged treatment time. Compared to the HUVECs treated with free CA4P for 24 h, the proliferation inhibition was attenuated by loading CA4P in the scaffolds. Nevertheless, due to the controlled release, CA4P@S gained a competitive advantage over free CA4P after the treatment for 48 h. A similar variation was also presented in the calcein acetoxymethyl ester (calcein‐AM/propidium iodide (PI)‐stained live/dead fluorescence images (Figure 2b). Additionally, a positively concentration‐correlated suppression of HUVEC proliferation was revealed by the live/dead staining for the CA4P both under free state and in the scaffolds, which indicated that the two selected CA4P concentrations fell within a rational range, warranting further investigation.^[^ ^7^, ^20^ ^]^ We also found the concurrence of negligible dead cells and large numbers of live cells in each fluorescence image of HUVECs treated with CA4P, which confirmed the previous findings that CA4P mainly restrained cell proliferation and rarely led to cell death.^[^ ^21^ ^]^ Regarding this confirmation, we conducted cell colony formation assays to investigate the CA4P@S‐caused proliferation suppression of HUVECs further. As shown in Figure 2c,d, the HUVEC colony number was significantly reduced after the coincubation with CA4P@S for 7 d. After the treatment with CA4P@S, the relative HUVEC colony‐forming rate decreased by more than 50.0%, and a more pronounced change occurred with the incremental CA4P concentrations. The long‐lasting colony formation inhibition also demonstrated the controlled release of CA4P from the scaffolds.

To clarify the latent mechanism for the CA4P@S‐caused proliferation inhibition, we investigated the influence of CA4P@S on the cell division and cell cycle of HUVECs. After the coincubation with CA4P@S for 12 h, the microtubes of HUVECs were stained with green fluorescence‐labeled tubulin antibody, and the representative confocal laser scanning microscope (CLSM) images and the corresponding quantifications were presented in Figure 2e,f, respectively. As the main components of the cytoskeleton, the microtubes in HUVECs without treatments or treated with blank scaffolds were stable enough to support the typical cell shapes (Figure 2e). With the introduction of CA4P, the intact microtubes disappeared, and the tubulin‐positive (tubulin^+^) fluorescence area per cell notably decreased (Figure 2f). Moreover, two separated nuclei could be observed in one HUVEC after the treatment with scaffolds containing CA4P (Figure 2e). The suppression of cell division was consistent with previous reports about CA4 or CA4P.^[^ ^20^ ^]^ CA4 is a binding agent for tubulins that can dynamically disaggregate and aggregate to participate in the formation of cytoskeletons or spindle apparatuses during an entire cell cycle.^[^ ^6^, ^7^, ^22^ ^]^ Thus, CA4P@S prevented the reaggregation of tubulins disaggregated from the spindle apparatuses in dividing HUVECs. As a result, the CA4P@S‐treated HUVECs were blocked in the G2/M phase during mitosis, as evidenced by the flow cytometric analysis based on PI staining (Figure 2g). The summarized quantification of cell cycle phases is shown in Figure 2h. Because of the treatment with CA4P@S, both the G0/G1 and S phase distribution percentages significantly decreased by about 20.0%, while the G2/M phase distribution percentage remarkably increased by ≈30.0%. Besides, the variations became more evident after the introduction of more CA4P (Figure 2h). Collectively, CA4P@S could suppress cell division by blocking mitosis, thus inhibiting the proliferation of VECs.

### 2.3 In Vitro Angiogenesis Suppression and Blood Vessel Destruction by CA4P@S

**Figure 3:**
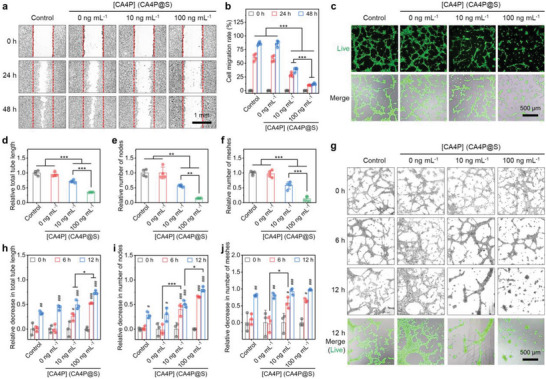
In vitro suppressive and destructive effect of CA4P@S on blood vessels. Inhibition of cell migration indicated by a) the postscratch bright‐filed images and b) quantified cell migration rates of HUVECs coincubated with CA4P@S for various durations. Suppression of angiogenesis reflected by c) the calcein fluorescence images and the relatively quantified d) total tube length, e) node number, and f) mesh number of capillary tubes formed by HUVECs coincubated with FGF2 and CA4P@S for 6 h. Destruction of preexisting blood vessels revealed by g) the bright‐filed or calcein fluorescence images and the relatively quantified h) total tube length, i) node number, and j) mesh number of capillary tubes pre‐established by the FGF2‐treated HUVECs and further treated with CA4P@S for different durations. Control group, HUVECs without the coincubation with CA4P@S. Data are shown as mean ± SD (*n* = 4 in panel (b), (d), (e), and (f); *n* = 3 in panel (h), (i), and (j)). Statistical significance was assessed by two‐way ANOVA in panels (b), (h), (i), and (j), and by one‐way ANOVA in panels (d), (e), and (f). *P < 0.05, **P < 0.01, and ***P < 0.001. In panels (h), (i), and (j), #*P* < 0.05, ##*P* < 0.01, and ###*P* < 0.001 versus the data from coincubation for 0 h in each group.

Angiogenesis in tumors is a complex bioprocess commonly involving the degradation of preexisting extracellular matrices, the migration and proliferation of VECs, the formation of new ductal branches and vascular rings, the reorganization of basement membranes, and the establishment of new lumens and tubes.^[^ ^23^ ^]^ Thus, the formation of new capillaries can be obstructed by VEC migration suppression. CA4P shows great potential to inhibit VEC proliferation and impair VEC motility.^[^ ^6^, ^24^ ^]^ To assess the effect of CA4P@S on the motility of VECs in vitro, a wound healing assay was performed on HUVECs. The results (**Figure** **3**a) showed that the wound gap between HUVECs in both the control (without any coincubations) and the treated groups gradually decreased as the incubation time increased. The semiquantifications of cell migration rates in Figure 3b show that the HUVEC wounds healed significantly slower with the introduction of CA4P. After the coincubation with CA4P@S for 24 or 48 h, the cell migration rate for HUVECs treated with 10 and 100 ng mL^−1^ CA4P was about half and a quarter of that for HUVECs treated without CA4P, respectively. Hence, the HUVEC migration interference also displayed remarkably positive dependence on the CA4P concentrations. The reason for this phenomenon might be the excellent ability of CA4P to prevent the reorganization of intracellular microtubules.^[^ ^24a^ ^]^

To directly evaluate the effect of CA4P@S on angiogenesis in vitro, we simulated the capillary network formation process by culturing HUVECs simultaneously with fibroblast growth factor 2 (FGF2) and CA4P@S on matrix gels. As presented in Figure 3c, the introduction of CA4P resulted in fewer capillary networks formed by HUVECs, and the downtrend exhibited a positive correlation with the CA4P concentrations. In addition, the relative quantifications for the total length of tubes and the number of nodes or meshes also demonstrated a corresponding significant reduction. After the treatment with the scaffolds containing 100 ng mL^−1^ CA4P in the culture medium, the inhibition rate of angiogenesis in vitro far exceeded 50.0% (Figure 3d–f). These results suggested that CA4P@S could effectively block the generation and development of neovascular capillaries during tumor growth. We subsequently estimated the effect of CA4P@S on the mature vascular networks in vitro using the HUVECs pretreated with FGF2. The microscopic images in Figure 3g showed that the preformed capillary networks were conspicuously destroyed after the co‐incubation with CA4P@S, and the longer incubation resulted in more damage. The relatively quantified results were displayed in Figure 3h–j, which demonstrated notable positive correlations between the CA4P concentrations and the decreases in the tube length, node number, and mesh number of vascularized HUVECs subjected to various treatments. After coincubation for 6 h, the CA4P@S with 10 and 100 ng mL^−1^ in the culture medium both already destroyed around 50.0% of the preexisting blood vessels in vitro. When the treatment was given for 12 h, the destruction rate almost reached 100.0%. Besides, the three capillary formation indices of HUVECs incubated without CA4P@S also declined with the prolonged culture after the establishment of capillary networks (Figure 3h–j). Perhaps, as HUVECs consumed FGF2 continuously, they might become less capable of building vessels. Consequently, CA4P@S showed the potential to destroy the preestablished blood vessels in tumor tissues. Furthermore, we used calcein‐AM to stain the live HUVECs and found that CA4P@S barely induced HUVEC death during the coincubation with or without the FGF2‐activated capillary network preformation. Combined with the results in Figure 2, CA4P@S could block the angiogenesis and disrupt the blood vessels not by killing VECs but via destabilizing their cytoskeletons, as reported previously.^[^ ^24a^ ^]^

### 2.4 In Vitro Mitochondrial Dysfunction and ROS Overaccumulation by Sandwich‐Structured Composites Containing TPZ in Interior Layer

**Figure 4:**
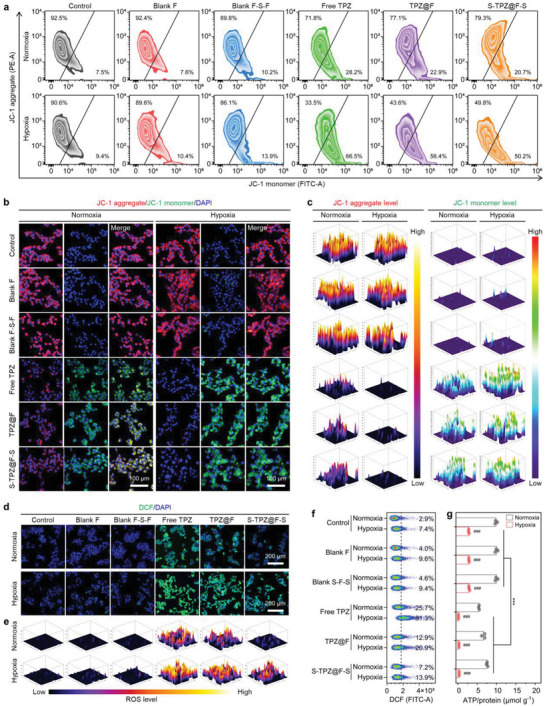
In vitro mitochondrial dysfunction and redox homeostasis disruption in cancer cells treated by TPZ in various states. Variations of ΔΨm reflected by a) the flow cytometry analysis, b) fluorescence images, and c) 3D surface plots indicating fluorescence intensity and distribution of JC‐1‐stained RM‐1 cells after the coincubation with different forms of TPZ for 12 h. Intracellular ROS levels represented by d) the fluorescence images, e) 3D surface plots, and f) flow cytometry analysis of DCF‐stained (ROS‐indicated) RM‐1 cells treated with various forms of TPZ for 12 h. In all fluorescence images, 4’,6‐diamidino‐2‐phenylindole dihydrochloride (DAPI) was used to stain the cell nuclei. g) ATP levels in RM‐1 cells undergoing coincubation with TPZ in various states for 24 h. Control group, RM‐1 cells without the coincubation with any forms of TPZ. Data are presented as mean ± SD (*n* = 3). Statistical significance in panel g was evaluated by two‐way ANOVA. ***P < 0.001. ###*P* < 0.001, by the intragroup comparison.

TPZ could be bioreduced to the free radical BTZ in the hypoxic intracellular environment.^[^ ^15^ ^]^ Both TPZ and BTZ could disrupt mitochondria via reducing the mitochondrial membrane potential (ΔΨm).^[^ ^25^ ^]^ Meanwhile, the level‐enhanced ROS produced by the TPZ bioreduction and dysfunctional mitochondria could attack nuclei, mitochondria, and other subcellular structures. Thereby, the internalized TPZ could induce mitochondrial dysfunction and oxidative damage in cancer cells under hypoxic conditions. To verify the cytotoxicity of TPZ in the electrospun fibers or sandwich‐like composites, we used two cytomembrane‐permeable fluorescence probes 5,5’,6,6’‐Tetrachloro‐1,1’,3,3’‐tetraethyl‐imidacarbocyanine iodide (JC‐1) and 2’,7’‐dichlorodihydrofluorescein diacetate (DCFH‐DA) to indicate the ΔΨm changes and ROS levels in RM‐1 cells with different treatments, respectively. As a cationic dye, JC‐1 could aggregate on the mitochondrial membranes with high ΔΨm and emit red fluorescence. While in the mitochondrion‐damaged cells with low ΔΨm, JC‐1 could remain in the monomeric state and fluoresce green.^[^ ^7b^ ^]^ The flow cytometry analyses of JC‐1‐stained RM‐1 cells were shown in **Figure** **4**a, from which we found that the JC‐1 aggregate^+^ proportions decreased, and the JC‐1 monomer^+^ proportions increased for the RM‐1 cells in both normoxic and hypoxic environments after the introduction of TPZ. Differently, for the RM‐1 cells treated with free TPZ, TPZ@F, and sandwich‐structured composites containing TPZ on fibers (S‐TPZ@F‐S), the JC‐1 aggregate^+^ proportions (71.8%, 77.1%, and 79.3%) under normoxia were about twice higher than those (33.5%, 43.6%, and 49.8%) under hypoxia, respectively. The JC‐1 monomer^+^ proportions (28.2%, 22.9%, and 20.7%) under normoxia were approximately one half of those (66.5%, 56.4%, and 50.2%) under hypoxia, respectively. Correspondingly, the aggregate/monomer ratios of mean fluorescence intensities for the RM‐1 cells after different treatments displayed analogous variations (Figure S4, Supporting Information). The fluorescence images (Figure 4b) of JC‐1‐dyed RM‐1 cells and the corresponding 3D‐surface plots (Figure 4c) both exhibited similar results, in which the introduction of TPZ attenuated the JC‐1 aggregate (red) fluorescence distribution areas and intensities. At the same time, contrary variations were observed for the JC‐1 monomer (green) fluorescence. These trends implied that TPZ could be successfully released from the fibers or sandwich‐structured composites during the coincubations, then leading to the mitochondrial dysfunction through diminishing the ΔΨm. The RM‐1 cells coincubated with S‐TPZ@F‐S showed the weakest JC‐1 monomer fluorescence and the most robust JC‐1 aggregate fluorescence among the three groups treated with TPZ in different forms, which suggested that the sandwiching could achieve the sustained and controlled release of TPZ, thereby ensuring its long‐term effective bioavailability after one implantation at the tumor resection site.

Besides lowering the ΔΨm, the introduced TPZ and bioreduced BTZ also promoted the level of ROS in RM‐1 cells (Figure 4d–f), which was the consequence of mitochondrial dysfunction as well. As an intracellular ROS indicator, DCFH‐DA can be rapidly hydrolyzed and oxidized to 2’,7’‐dichlorofluorescein (DCF), emitting strong green fluorescence in the cells with high ROS levels. As illustrated in the microscopic images (Figure 4d) and the 3D‐surface plots (Figure 4e), the DCF fluorescence distribution areas and intensities in RM‐1 cells were evaluated by the coincubation with TPZ in various forms, and the hypoxic environments resulted in a higher level of intracellular ROS than normoxic environments. Similar results were presented by the flow cytometry analyses of DCF‐stained RM‐1 cells in Figure 4f. After the treatment with free TPZ, TPZ@F, and S‐TPZ@F‐S, the DCF^+^ proportions of RM‐1 cells increased from 2.9% to 25.7%, 12.9%, and 7.2%, respectively, under normoxic conditions. The incubation under hypoxia led to greater rises in the corresponding DCF^+^ proportions, which increased from 7.4% to 81.3%, 20.9%, and 13.9%, respectively. The S‐TPZ@F‐S‐treated RM‐1 cells generated the least ROS compared to the groups treated with free TPZ or TPZ@F, well matching the JC‐1 staining results and confirming the controlled release of TPZ. The abundances of ATP produced by mitochondria in RM‐1 cells significantly decreased by about 5.0 fold after the treatment with different forms of TPZ in hypoxic environments (Figure 4g). This result could be explained by the TPZ‐ or BTZ‐induced damages on mitochondria and the reciprocal causation between ROS overaccumulation and mitochondrial dysfunction. Therefore, the TPZ controllably released from S‐TPZ@F‐S could cause oxidative damage in cancer cells and effectively obstruct the intracellular energy supply through mitochondrial dysfunction.

### 2.5 In Vitro Apoptosis Promotion and Metastasis‐Associated Protein Downregulation by S‐TPZ@F‐S

**Figure 5:**
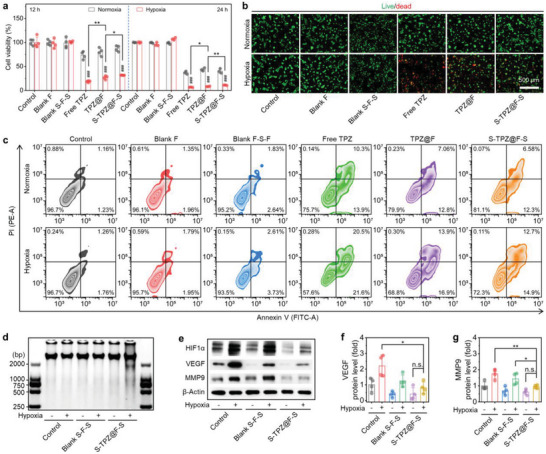
In vitro apoptosis and inhibition of metastasis‐associated gene for cancer cells treated with different forms of TPZ. a) Cell viability of RM‐1 cells co‐incubated with TPZ in various states for different durations. b) Calcein‐AM/PI (live/dead)‐stained fluorescence images and c) apoptosis flow cytometry analysis of RM‐1 cells treated with TPZ in various states for 12 h. d) Agarose gel electrophoresis images of DNA, and e) western blots and f,g) the relative quantification of characteristic proteins in RM‐1 cells undergoing the coincubation with different forms of TPZ for 12 h. Control group, RM‐1 cells without the coincubations with any forms of TPZ. Data are presented as mean ± SD (*n* = 4 for the 12 h of coincubation in panel (a), and in panels (f) and (g); *n* = 3 for the 24 h coincubation in panel (a)). Statistical significance was estimated by two‐way ANOVA in panel (a), and by one‐way ANOVA in panels (f) and (g). *P < 0.05, **P < 0.01, and ***P < 0.001. In panel (a), ###*P* < 0.001, by the intragroup comparison.

Considering the intracellular broken redox homeostasis and dysfunctional mitochondria, we further detected the cytotoxicity and bioprocess regulations induced by S‐TPZ@F‐S. The CCK8 results (**Figure** **5**a) showed that the used electrospun fibers and sandwich‐like composites exhibited extremely low cytotoxicity, as evidenced by the almost 100.0% viability of RM‐1 cells after the coincubations for 12 or 24 h under normoxic or hypoxic conditions. As expected, the viabilities of RM‐1 cells treated with various states of TPZ all decreased. The decreases became more pronounced when the bioreduction of TPZ to BTZ occurred specifically in hypoxic environments. After the treatment with free TPZ, TPZ@F, or S‐TPZ@F‐S for 12 or 24 h, the corresponding RM‐1 cell viability under hypoxia was extremely significantly lower than that under normoxia. Caused by the controlled release, the loading in fibers sandwiched by two scaffolds reduced the cytotoxicity of TPZ at the same detection time point, consistent with the ΔΨm and ROS fluorescence analyses of treated RM‐1 cells (Figure 4). As shown in Figure 5b, the live/dead fluorescence images visually illustrated similar trends to CCK8 results. There were few dead cells signified by red fluorescence in the representative fluorescence images of RM‐1 cells coincubated without TPZ for 12 h in normoxia or hypoxia. The introduction of TPZ resulted in greatly elevated PI‐stained dead cells, especially after the hypoxic cultures. Indubitably, with the loading in fibers and sandwiching by scaffolds, the controllably released TPZ resulted in less cell death (Figure 5b). Moreover, we carried out the apoptosis flow cytometry on RM‐1 cells with various treatments for 12 h, and the corresponding analyses are shown in Figure 5c. It was found that the early (annexin V‐FITC^+^PI^−^) and late (annexin V‐FITC^+^PI^+^) apoptosis proportions of RM‐1 cells after the coincubation without TPZ for 12 h in normoxia or hypoxia were almost equal and low, all ranging from 0.0% to 4.0%, again demonstrating the low cytotoxicity of used materials. Certainly, the early and late apoptosis proportions of RM‐1 cells were respectively evaluated after the addition of free TPZ (10.3% and 13.9%), TPZ@F (7.1% and 12.8%), and S‐TPZ@‐S (6.6% and 12.3%) under normoxia. In addition, the hypoxic environments led to relatively higher corresponding proportions of RM‐1 cells treated with free TPZ (20.5% and 21.6%), TPZ@F (14.0% and 16.9%), and S‐TPZ@‐S (12.7% and 14.9%). Based on these results, we deduced that S‐TPZ@‐S could controllably release TPZ as expected and continuously excrete the hypoxia‐responsive cytotoxicity of TPZ.

Afterward, the anticancer mechanisms of S‐TPZ@‐S that kill RM‐1 cells and inhibit tumor metastasis were further explored. The DNA gel electrophoresis results are presented in Figure 5d, in which only the DNA bands of RM‐1 cells coincubated with S‐TPZ@‐S under hypoxia displayed an elongated and dispersive distribution, demonstrating the existence of numerous DNA fragments. These phenomena indicated that the released and internalized TPZ could be bioreduced and interact with the nuclear DNA and destroy the DNA chains.^[^ ^26^ ^]^ Although the in vitro experiments on HUVECs confirmed that the CA4P in the outer layers of used sandwich‐like composites could damage the preestablished blood vessels and suppress angiogenesis, further blocking the supply of nutrition and oxygen to restrain the tumor development. However, the progressively aggravate hypoxia could upregulate HIF1*α*‐VEGF pathway and favor the neovascularization, which was detrimental to the postsurgical tumor management.^[^ ^9^ ^]^ The western blots (Figure 5e) and the corresponding relative quantifications (Figure 5f) verified that the hypoxic culture endowed RM‐1 cells with higher HIF1*α* and VEGF abundances than the normoxic culture. After the treatment with S‐TPZ@‐S, the HIF1*α*‐VEGF pathway, especially the VEGF expression in RM‐1 cells incubated under hypoxic conditions, was remarkably inhibited, suggesting that TPZ could prevent the intercellular biosynthesis of HIF1*α*. Thus, the TPZ in the inner layers of used sandwich‐structured composites could counteract the unfavorable effect of the first‐released CA4P. This process guaranteed the intensified hypoxia necessary for the bioreduction to produce cytotoxic BTZ and facilitated the starvation therapy of CA4P. We also used a western blot assay to determine the intracellular level of MMP9, another HIF1*α* downstream protein, which could be oxidatively degraded.^[^ ^9a^ ^]^ As reported in earlier studies, the high expression and secretion of MMP9 could conduce to tumor invasion and metastasis.^[^ ^27^ ^]^ Unsurprisingly, the MMP9 in RM‐1 cells were upregulated at the lowered oxygen concentration, exhibiting a similar uptrend to HIF1*α* and VEGF. Following the introduction of TPZ, the intracellular MMP9 level significantly decreased, especially in hypoxic environments. Thereby, the CA4P preferentially released from scaffolds could inhibit cancer cell migration by blocking or cutting off external channels, i.e., blood vessels. The TPZ subsequently released from fibers could consolidate neovascularization inhibition and prevent cancer cell migration and colonization by downregulating MMP9. This dual‐drug‐loaded sandwich‐structured implant thus had exciting potential to suppress tumor metastasis.

### 2.6 In Vivo Postsurgical Tumor Recurrence Inhibition by CA4P@S‐TPZ@F‐CA4P@S

**Figure 6:**
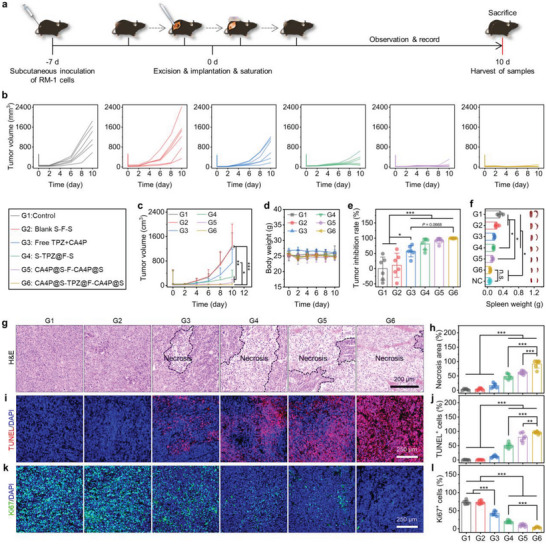
In vivo inhibition of postoperative tumor recurrence. a) Schematic diagram of experimental procedure involving RM‐1 tumor‐bearing mouse model establishment, tumor excision, subcutaneous implantation, and sample harvest. b) Individual and c) average tumor volume variations, and d) average body weight changes over time for the mice in different treatment groups. e) Tumor inhibition rate calculated based on the weights of PFA‐fixed tumors, and f) weights and the representative photographs of PFA‐fixed spleens from mice undergoing different treatments (NC = normal control). Representative microscopic images of tissue sections stained with g) H&E, i) TUNEL fluorescence, and k) Ki67 immunofluorescence for the tumors in different groups. Quantifications of h) necrosis area, j) TUNEL^+^ cells, and l) Ki67^+^ cells based on the captured images of tumor slices from mice subjected to various treatments. Data are presented as mean ± SD (*n* = 6 in panels (c), (d), (e), (h), (j), and (l); *n* = 4 in panel (f). Statistical significance was evaluated by two‐way ANOVA in panel (c), and by one‐way ANOVA in panels (e), (f), (h), (j), and (l). *P < 0.05, **P < 0.01, and ***P < 0.001.

After separately demonstrating the angiogenesis suppression, vascular disruption, mitochondrial dysfunction, ROS overproduction, hypoxia‐responsive cytotoxicity, and tumor metastasis prevention effects of CA4P@S‐TPZ@F‐CA4P@S in vitro, we used RM‐1 tumor‐bearing C57BL/6 mice to develop a tumor‐partially‐resected animal model to further investigate the postsurgical tumor recurrence and metastasis inhibition effect of CA4P@S‐TPZ@F‐CA4P@S in vivo. The experimental procedure is shown in **Figure** **6**a. One week after the subcutaneous inoculation of suspended RM‐1 cells within the logarithmic growth phase, about 90% of each tumor was excised with a remaining volume of 40–50 mm^3^ to simulate the clinical tumor‐positive margins after resection. According to the postoperative therapy methods, mice were divided into 6 groups, i.e., control (the group without any adjuvant treatments), blank S‐F‐S (the group implanted with blank sandwich‐like composites at the operative sites), free TPZ+CA4P (the group undergoing the injection of PBS containing TPZ and CA4P into the residual tumor tissues), S‐TPZ@F‐S (the group implanted with S‐TPZ@F‐S), CA4P@S‐F‐CA4P@S (the group implanted with sandwich‐structured composites containing CA4P in the exterior scaffolds), CA4P@S‐TPZ@F‐CA4P@S (the group implanted with CA4P@S‐TPZ@F‐CA4P@S). During the following postsurgical observation, the tumor volumes and body weights were monitored and recorded every 2 days. When a tumor volume exceeded 1500.0 mm^3^, the treatment was halted, and all the mice were sacrificed to harvest their recurrent tumors, peripheral blood, and major organs for further investigations. As shown in Figure 6b,c, the tumor volumes in the groups of control, blank S‐F‐S, and free TPZ+CA4P progressively increased with the prolonged treatment time, and the uptrends became more dramatic from day 6 after surgeries. Notably, the free TPZ+CA4P inflicted little suppression on the tumor regrowth, which might be attributed to the rapid dispersion and metabolism of free drugs after the one‐shot injection.^[^ ^28^ ^]^ In marked contrast, due to sustained drug release, the CA4P‐ or TPZ‐loaded sandwich‐like implants pronouncedly prevented postoperative tumor recurrence. Compared to the initial tumor volumes on the operation day, the final average tumor volumes on day 10 after implantations decreased 44%, 80%, and almost 100.0% for the groups of S‐TPZ@F‐S, CA4P@S‐F‐CA4P@S, and CA4P@S‐TPZ@F‐CA4P@S, respectively. All treatments hardly impacted the body weights (Figure 6d), preliminarily suggesting the systemic biosafety of the in situ drug delivery based on our fabricated sandwich‐structured implants. To further compare the inhibitions of tumor recurrence, the weights of sampled and paraformaldehyde (PFA)‐fixed tumors from distinct groups were measured and used to calculate the tumor inhibition rates as per the method and formula described in the Experimental Section. As presented in Figure 6e, the tumor inhibition rates of both control and blank S‐F‐S groups were almost 0%, while the tumor inhibition rates of CA4P@S‐TPZ@F‐CA4P@S group were nearly 100.0%, higher than those of the free TPZ+CA4P group (close to 50%). The results suggested that the controlled and sequential drug release endowed the sandwich‐structured implants containing TPZ and CA4P with superior antitumor effects. Interestingly, the mice with larger or heavier tumors had larger spleens (Figure 6f). According to previous reports, the malignant tumor‐bearing mice can exhibit a leukemoid reaction caused by the colony‐stimulating factors that are generated by cancer cells. This special cancer‐associated reaction is characterized by bone marrow hyperplasia, a rapidly evolving granulocytosis, which can cause diverse hematopoietic precursor cells, including metamyeloblasts and megakaryoblasts, to infiltrate into the splenic red pulp, further inducing splenomegaly.^[^ ^29^ ^]^ Besides, there have also been reports stating that the body's immune system could be highly activated during tumor invasion and metastasis. The spleen could produce or recruit high numbers of lymphocytes and monocytes, thus causing splenomegaly.^[^ ^30^ ^]^ This phenomenon preliminarily verified that the RM‐1 cells we inoculated in mice successfully formed malignant metastatic tumors, in line with an earlier report.^[^ ^31^ ^]^ With the postoperative RM‐1 tumor recurrence inhibition by introducing CA4P or TPZ (Figure 6f), the spleen weights and sizes decreased significantly and returned to normal.

To further investigate the in vivo antitumor efficacy of the executed therapy, the tumor tissues from mice with different treatments were fixed, sectioned, and subjected to hematoxylin and eosin (H&E) staining (Figure 6g,h), TUNEL fluorescence staining (Figure 6i,j), and Ki67 immunofluorescence staining (Figure 6k,l). As expected, the pronounced pyknosis, fragmentation, or dissolution of nuclei or cells, reflecting the necroses, were found in the H&E‐stained images of tumor slices from the mice treated with CA4P or TPZ (Figure 6g). Especially after the treatment with dual‐drug‐loaded composites, the average percentage of necrosis areas in the captured microscopic images was close to 100.0% (Figure 6h). Meanwhile, the cell apoptosis levels of the tumors in various groups were assessed by the TUNEL staining, and the corresponding fluorescence images are presented and quantified in Figure 6i,j, respectively. In accordance with the above results, the groups of free TPZ+CA4P, S‐TPZ@F‐S, and CA4P@S‐F‐CA4P@S all exhibited relatively higher TUNEL^+^ (red, revealing apoptosis) cell proportions in tumors than the groups of control and blank S‐F‐S. The severest cell apoptosis indicated by the largest red fluorescence area was observed in the representative image of tumor sections from the CA4P@S‐TPZ@F‐CA4P@S group (Figure 6i), of which the average TUNEL^+^ cell percentage in tumors was the highest and nearly 100.0% (Figure 6j). The cell proliferations of the harvested tumors were detected with the Ki67 immunofluorescence staining. As seen in Figure 6k,l, the cell proliferation in tumors was diminished by CA4P or TPZ. The number of Ki67^+^ (green) cells in the tumors from the mice implanted with CA4P@S‐TPZ@F‐CA4P@S was the smallest, and the corresponding average Ki67^+^ (green) cell percentage was the lowest and nearly 3.0%. Collectively, the in vivo antitumor therapy results supported the in vitro findings and inferences, demonstrating that CA4P@S‐TPZ@F‐CA4P@S efficiently inhibited the postsurgical tumor recurrence.

### 2.7 In Vivo Postoperative Tumor Metastasis Suppression by CA4P@S‐TPZ@F‐CA4P@S

**Figure 7:**
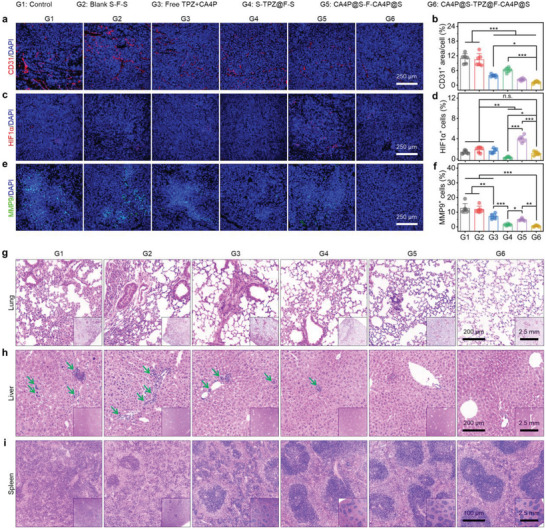
In vivo suppression of postsurgical tumor metastasis. Representative immunofluorescence images of tissue sections indicating the levels of a) CD31, c) HIF1*α*, and e) MMP9 in RM‐1 tumors from the mice undergoing different treatments. Quantifications of b) CD31^+^ cells, d) HIF1*α* ^+^ cells, and f) MMP9^+^ cells based on the fluorescence images of RM‐1 tumor slices in different groups. Representative H&E‐stained microscopic images of g) lung, h) liver, and i) spleen slices from the mice with different treatments. Green arrows indicate metastatic focus. Data are presented as mean ± SD (*n* = 6). Statistical significance in panels (b), (d), and (f) was evaluated by one‐way ANOVA. *P < 0.05, **P < 0.01, ***P < 0.001, and n.s., no significance.

After confirming that CA4P@S‐TPZ@F‐CA4P@S prevented the RM‐1 tumor regeneration in vivo, we further probed the efficacy and mechanism of tumor metastasis suppression by CA4P@S‐TPZ@F‐CA4P@S based on the sampled tumor tissues. As shown in **Figure** **7**a,b, the introduction of CA4P significantly impaired the intratumoral vascularity, evidenced by the reduced CD31^+^ (red) fluorescence in the representative microscopic image and the lowered average CD31^+^ area per cell calculated based on the captured images of tumor sections from the mice undergoing CA4P‐associated therapies. While the O_2_ supply obstruction by CA4P could favor the hypoxia‐responsive cytotoxicity of TPZ, the intracellular upregulated HIF1*α* (Figure 7c,d) might stimulate the expression and secretion of VEGF. The upregulation of VEGF was conducive to VEC maturation and neovascularization (Figure 7c,d), which could limit intratumoral hypoxia enhancement and disfavor starvation therapy.^[^ ^7^, ^32^ ^]^ Thus, it was important to introduce TPZ. As presented in Figure 7c,d, the internalized TPZ downregulated the intratumoral HIF1*α*, illustrated by the scarce HIF1*α* ^+^ (red) fluorescence in the representative image and the lowest average HIF1*α* ^+^ cell proportion (close to 0.0%) calculated based on the acquired images of tumor slices from the mice treated with S‐TPZ@F‐S. This finding is consistent with the in vitro western blot results (Figure 5e,f). Moreover, the combined treatment of TPZ and CA4P not only neutralized the adverse effect of CA4P monotreatment but also consolidated the CA4P‐induced angiogenesis inhibition and preexisting blood vessel destruction. The HIF1*α* ^+^ cell proportions of tumor slices from the groups treated with the CA4P‐contained implants were remarkably reduced by the subsequently released TPZ (Figure 7c,d). The CA4P@S‐TPZ@F‐CA4P@S group also displayed less CD31^+^ area in the representative tumor section image than CA4P@S‐F‐CA4P@S group (Figure 7a,b). After loading in the sandwich‐like composites, the drug combination exerted a greater inhibitory effect on the intratumoral blood vessels, which more effectively cut off the cancer cell migration channels. In addition, we also investigated the intratumoral level of MMP9, a crucial secretory protease with the capability to degrade the extracellular matrix and destroy the physical barriers of tumor invasion and metastasis.^[^ ^33^ ^]^ In accordance with the results of western blot experiments performed on RM‐1 cells (Figure 5e,g), the MMP9^+^ (green) cell proportions in the images of tumor slices were significantly lessened by the TPZ‐associated treatments (Figure 7e,f). The ROS‐sensitive MMP9 could be inactivated or degraded after the internalization and bioreduction of TPZ. Unexpectedly, the CA4P monotreatment also reduced the intratumoral MMP9 abundance. Perhaps, the external energy obstruction could heighten intracellular ROS levels.

According to the previous study, we used H&E to stain the tissue sections of the lungs and livers from mice to visualize the RM‐1 tumor metastasis.^[^ ^31c^ ^]^ The representative images are displayed in Figure 7g,h, respectively. Compared to the control and blank S‐F‐S groups, both the metastatic tumor nodule numbers and areas in the lungs and livers decreased in the drug‐administered groups. The metastases were invisible in the tissue slices of lungs and livers from the CA4P@S‐TPZ@F‐CA4P@S group. As well known, the spleen as a vital immune organ can generate lymphocytes and monocytes, forming the white pulps. Congruous with the variations in weights and sizes of spleens (Figure 6g), due to the continuous development of RM‐1 tumors, the protective immune response was excessively activated. Specifically, the cancer‐associated leukemoid reaction‐induced granulocytic infiltrates and the overproduction and recruitment of lymphocytes and monocytes resulted in the indistinct boundaries of red and white pulps in spleens (Figure 7i).^[^ ^29^, ^30^ ^]^ After the treatment with CA4P or TPZ, the boundaries in spleens returned to baseline due to the inhibition of tumor recurrence and metastasis. The most apparent boundaries are presented in the representative image of spleen slices from the mice undergoing the implantation with CA4P@S‐TPZ@F‐CA4P@S (Figure 7i). Therefore, the dual‐drug treatment with CA4P and TPZ suppressed the postoperative tumor metastasis, and the sequent and controlled release from the sandwich‐structured implants substantially improved the therapeutic efficacy.

### 2.8 Transcriptome Analysis Revealing Therapeutic Mechanisms of CA4P@S‐TPZ@F‐CA4P@S on Postsurgical Tumor Recurrence and Metastasis

**Figure 8:**
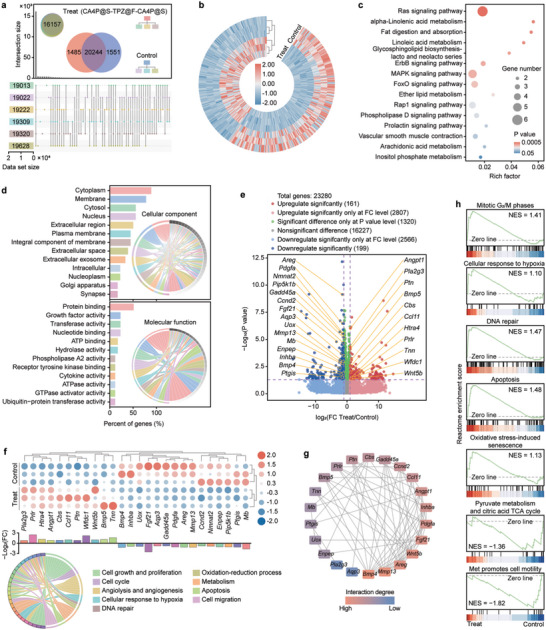
Transcriptome analysis based on RNA sequencing of RM‐1 tumors from mice with different treatments. a) Upset plot with Veen diagram showing the number and intersection of genes among the samples from control and CA4P@S‐TPZ@F‐CA4P@S groups. b) Heatmap reflecting significantly (FC > 2 and *P* < 0.05) regulated DEGs, and enrichment analyses of c) KEGG pathway and d) GO terms of DEGs among the samples from different groups. e) Volcano plot representing upregulated and downregulated DEGs (significant regulation only at FC level, FC > 2 and *P* > 0.05; significant regulation only at *P* value level, FC < 2 and *P* < 0.05; nonsignificant difference, FC < 2.0 and *P* > 0.05) among the tumors from mice with different treatments. f) Heatmap with pathway enrichment and g) PPI network of significantly regulated DEGs involving tumor recurrence and metastasis (especially presented in volcano plot). h) GSEA based on the Reactome pathway enrichment analysis showing upregulated or downregulated pathways [absolute value of normalized enrichment score (|NES|) > 1] associated with the proposed tumor treatment mechanisms for CA4P or TPZ in the sandwich‐structured implants.

To consolidate the results and further verify the corresponding inferences, we performed RNA sequencing and transcriptome analysis on six randomly selected tumor samples, including three from the control group and three from the CA4P@S‐TPZ@F‐CA4P@S group. According to the acquired gene expression matrix (GEM), the principal component analysis (PCA) was first conducted, and a noticeable difference was observed between the transcriptomes from the two tested groups (Figure S5a, Supporting Information). Then, further analyses based on GEM were performed. The obtained upset plot with Veen diagram (**Figure** **8**a) illustrated that the selected six samples and two groups shared 16 157 and 20 244 coexpressed genes, respectively. Meanwhile, 1485 and 1551 genes were exclusively expressed by the control group and CA4P@S‐TPZ@F‐CA4P@S group, respectively. The heatmap of differentially expressed genes (DEGs) identified as the genes with absolute fold change (FC) > 2.0 and *P* < 0.05 are presented in Figure 8b, displaying the upregulated (red) and downregulated (blue) DEGs between the two groups. Based on the DEGs, the enrichment analyses involving the Kyoto encyclopedia of genes and genome (KEGG) pathways and gene ontology (GO) terms were carried out to explain better how CA4P@S‐TPZ@F‐CA4P@S suppress the postoperative tumor recurrence and metastasis. The top 15 significantly enriched pathways were enumerated in the KEGG pathway bubble plots (Figure 8c), of which the 4 most significantly regulated signaling pathways were RAS, MAPK, FOXO, and ERBB, respectively. As reported in the previous studies and presented in Figures S6–S9 (Supporting Information), the four signaling pathways mainly involved cell growth, cell cycle, cell proliferation, oxidative stress, DNA repair (correcting response to the DNA alteration or damage), metabolism, apoptosis, migration, invasion, and angiogenesis, all of which were strongly associated with the tumor progress or inhibition and verified the CA4P@S‐TPZ@F‐CA4P@S antitumor mechanisms inferred from the in vitro and in vivo results. The GO enrichment analyses are shown in Figure S5b (Supporting Information) and Figure 8d, demonstrating that compared to the control group, the postsurgical treatment with CA4P@S‐TPZ@F‐CA4P@S changed the biological process, cellular component, and molecular function. Most of the altered GO terms were also related to cell growth, proliferation, metabolism, and migration. Besides, as presented in the volcano plot (Figure 8e), among the 23 280 total genes in tumors of the two groups, 161 genes and 199 genes were significantly upregulated and downregulated by the therapy with CA4P@S‐TPZ@F‐CA4P@S, respectively. Especially, 26 significantly regulated genes were indicated, of which the detailed variations in all samples were displayed in a heatmap with a simple enrichment graph (Figure 8f). The 26 DEGs were mainly involved in the cell growth and proliferation, cell cycle, angiolysis and angiogenesis, cellular response to hypoxia, DNA repair, oxidation‐reduction process, metabolism, apoptosis, and migration, which were broadly in line with the bulk enrichment analyses of KEGG pathways and GO terms and suggested a new enrichment under hypoxia‐related changes. Moreover, the protein–protein interaction (PPI) network was graphed in Figure 8g to reflect the functional interactions among proteins translated from the representative DEGs. The red color represents the high interaction degree and importance of the corresponding gene in the PPI network. The Bmp4, Mmp13, Areg, Wnt5b, Fgf21, and Pdgfa genes played the most crucial roles, which were mainly involved in cell growth and proliferation, angiolysis and angiogenesis, and migration and invasion (Figure 8f).^[^ ^34^ ^]^

To further confirm the mechanisms for the preventive effect of CA4P@S‐TPZ@F‐CA4P@S on postoperative tumor recurrence and metastasis, we performed the gene set enrichment analysis (GSEA) based on the Reactome enrichment of DEGs, and the analysis results were plotted in Figure 8h; and Figure S10 (Supporting Information). The GSEA plots in Figure 8h showed positive enrichments of the gene signatures involved in the mitotic G_2_/M phase (inhibition of cell cycle), cellular response to hypoxia, DNA repair, apoptosis, and oxidative stress‐induced senescence and negative enrichments of the gene signatures concerned with pyruvate metabolism and citric acid TCA cycle (obstruction of the intracellular energy supply) and cell motility promoted by Met. The GSEA plots in Figure S10 (Supporting Information) were basically in agreement with the results in Figure 8. Surprisingly, the DEGs engaged in innate and adaptive immune systems were positively enriched (Figure S10, Supporting Information), which demonstrated the probable immunogenetic cell death or direct antitumor immune response caused by the implantation of CA4P@S‐TPZ@F‐CA4P@S. This interesting GSEA result meant more possibilities for the reinforced antitumor treatment with the sandwich‐structured implants carrying CA4P and TPZ, which deserved extended and in‐depth investigations. Collectively, the transcriptome analyses based on RNA sequencing of RM‐1 tumors further elucidated the antitumor mechanisms of CA4P@S‐TPZ@F‐CA4P@S.

### 2.9 Biosafety Evaluation

It was essential to evaluate the biosafety of the locally administered drugs and implanted composites. Besides the demonstration of neglectable changes in the mouse body weights during the duration of therapy, we also used histological and serological detections to further confirm the low toxic and side effects of the implemented treatments. As shown in Figure S11 (Supporting Information), there were few necroses or disorders in the representative H&E‐stained tissue section images of the hearts or kidneys from all groups, evidencing the hypo‐ or nontoxicity of in situ delivered CA4P or TPZ and the implants on the critical organs and normal tissues. Additionally, almost all the averages of tested serum biochemical indices, including alanine transaminase (ALT), aspartate aminotransferase (AST), albumin (ALB), alkaline phosphatase (ALP), urea, creatinine (CREA), creatine kinase MB (CKMB), and lactate dehydrogenase (LDH) were located at the corresponding recommended ranges of mice and implied the normal functions of livers (ALT < 27.2 U L^−1^, AST < 155.0 U L^−1^, ALB < 30.1 g L^−1^, and ALP < 119.0 U L^−1^), kidneys (Urea < 9.0 mm and CREA < 17.0 µm), and hearts (CKMB < 139.0 U L^−1^ and LDH < 1257.0 U L^−1^) in mice (Figure S12, Supporting Information), respectively, again validating the biosafety of the used treatments.^[^ ^7^, ^16^, ^35^ ^]^ It was worth mentioning that the AST levels in serums from 1–3 out of 6 mice implanted with blank S‐F‐S were higher than the suggested maximum values, which was perhaps caused by the invasion and metastasis of RM‐1 tumors.^[^ ^36^ ^]^

## 3 Conclusion

In summary, we constructed an implantable sandwich‐structured composite. The exterior layers are two 3D‐printed scaffolds loaded with CA4P, and the interior layer is a patch of electrospun fibers loaded with TPZ. Based on the in vitro, in vivo, and transcriptome sequencing results, we confirmed the implants could prevent RM‐1 tumor postsurgical recurrence and metastasis via blocking the energy supply and activating hypoxia‐initiated chemotherapy. After tumor resection and composite implantation, CA4P was preferentially released to destroy the intratumor preexisting blood vessels and inhibit neovascularization, which not only heightened the hypoxic level in TME but also cut off the external energy supply and migration channels of cancer cells. Subsequently, TPZ was released and bioreduced to cytotoxic BTZ under the hypoxia intensified by CA4P. On the one hand, the internalization and bioreduction of TPZ generated ROS, broke intracellular redox homeostasis, produced oxidative damage, disrupted mitochondria, and impeded the generation of intracellular ATP, i.e., obstructed the intracellular energy supply. On the other hand, BTZ directly attacked the cell nuclei and damaged the nuclear DNA. In addition, we also found that the reduction product BTZ lessened the intracellular abundances of HIF1*α*, VEGF, and MMP9. The downregulated HIF1*α* and VEGF strengthened the inhibitory effect of CA4P on angiogenesis, creating a closed‐loop reinforcement for the obstruction of extracellular energy supply and cancer cell migration. The downregulated MMP9 disfavored the degradation of the extracellular matrix, suppressing the migration and invasion of cancer cells. Therefore, our designed and fabricated sandwich‐like implants loaded with CA4P and TPZ could efficiently exert a dual‐drug combined therapeutic effect to inhibit tumor recurrence and metastasis, offering a facile strategy for neoadjuvant chemotherapy after the excision of solid tumors.

## 4 Experimental Section

### Materials

CA4P was purchased from RYON (Shanghai, China). TPZ, SA, CaCl_2_, and RhB were bought from Macklin (Shanghai, China). PLGA ([*η*] = 0.8 dL g^−1^) was obtained from Polymtek (Shenzhen, China). PVA (molecular weight = 67 000), petroleum ether (boiling point = 60–90 °C), penicillin G sodium, streptomycin sulfate, HFIP, and tween 20 were purchased from Aladdin (Shanghai, China). Defatted soybean meal defatted at a low temperature was obtained from Yuwang (Yucheng, China). Calcein‐AM, PI, DCFH‐DA, DAPI) RNase A, and skim milk powder were bought from Beyotime (Shanghai, China). Recombinant human FGF2 was obtained from Solarbio (Beijing, China). Matrigel (growth factor reduced and phenol red free) was purchased from BD (Franklin Lakes, USA). JC‐1 was bought from Bidepharm (Shanghai, China). All other reagents were analytically pure and used as received. Ultrapure water (18.2 MΩ cm^−1^) used in the study was prepared by a lab water purification system (Milli‐Q Integral 3, Millipore, USA).

### Preparation and Characterization of SPI—Preparation of SPI

SPI was prepared according to the previously reported procedures with slight modifications.^[^ ^17^ ^]^ Initially, the defatted soy meal was ground into finer powder by a high‐speed grinder (BJ‐800A, Baijie, China) for 4 cycles with 10 s on and 20 s off per cycle. Subsequently, the refined soy powder was redefatted with refluxed petroleum ether in a Soxhlet apparatus for 12 h at 70 °C. After complete drying in a hot air oven at 70 °C, the redefatted soy powder was dispersed in ultrapure water at a 1:20 weight/volume w/v ratio by a mechanical agitator (RW 20 digital, IKA, Germany) at 150 rpm. The pH was adjusted to 8.0 using an aqueous solution of NaOH (5 m). The mixture was constantly agitated for 2 h at room temperature, while the pH was readjusted to 8.0 using a 1 m NaOH solution every 20 min. After centrifugation (8000 g) for 30 min at 4 °C, the pH of the harvested supernatant was adjusted to 4.5 using a 5 M HCl solution. The resultant precipitates were collected by centrifugation (5000 g) for 30 min at 4 °C and redispersed in ultrapure water at a 1:5 w/v ratio by magnetic stirring. Then, the pH was adjusted to 7.0 to obtain a clear solution. The crude SPI solution was transferred into dialysis tubes with a cut‐off molecular weight of 8000–14 000 Da and dialyzed in stirred ultrapure water at a 1:30 volume/volume v/v ratio for 48 h at 4 °C, during which the water was refreshed every 12 h. Finally, the dialysate was collected and lyophilized, and the dried SPI was stored at −30 °C for further use.

### Preparation and Characterization of SPI—PAGE of SPI

To assess the purity of prepared SPI and investigate the variations in SPI subjected to the pretreatment for 3D‐printing ink preparation, the typical reducing and nonreducing PAGE were performed on native SPI and heated SPI with or without PVA. Before sample loading, the samples used for reducing PAGE were mixed with a 5 × dithiothreitol‐contained buffer solution (Beyotime) at a 1 : 4 v/v ratio and denatured through heating (95 °C) and oscillation (1200 rpm) for 30 min in a shaking incubator (MSC‐100, Thermo, USA), and the samples used for nonreducing PAGE were thoroughly mixed with a 5 × loading buffer solution without any reductants or denaturants (Beyotime) through a vortex for 3 min at room temperature. Subsequently, the reduced and nonreduced samples were separately loaded onto a prefabricated SDS‐free polyacrylamide gel composed of 4% stacking gel and 20% separating gel (Beyotime). The reducing and nonreducing electrophoreses were carried out in an SDS‐contained tris (hydroxymethyl) aminomethane (Tris)‐glycine (Gly) buffer system (Beyotime) and an SDS‐free Tris‐Gly buffer system (Beyotime), respectively. After PAGE, the gels were stained with 0.25% w/v Coomassie brilliant blue R250 in a methanol/acetic acid/water (v/v/v = 5:1:4) solution by shaking (60 rpm) for 3 h at room temperature, followed by destaining overnight in a solution containing 5% v/v methanol and 7.5% v/v acetic acid. The images of destained gels were acquired with a multifunctional imaging system (G: BOX Chemi XX6, Syngene, UK).

### Fabrication and Characterization of Sandwich‐Structured Composite Composed of Electrospun Fibers and 3D‐Printed Scaffolds—Fabrication of Electrospun Fibers

PLGA (10%, w/v) was completely dissolved in HFIP with or without TPZ (0.048%, w/v) by stirring overnight at room temperature, and the homogeneous solution was filled into an injector carrying a syringe needle with a diameter of 0.25 mm and fixed on the electrospinning device. After bubble exhaustion, the solution was fed at 0.3 mL h^−1^ into an electric field at a constant voltage of 12 kV, during which the distance between the needle and rolling collector was set as 10 cm. Following the adequate volatilization of solvent, the harvested fibers were stored in a desiccated environment for further use. The blank fibers without TPZ were named blank F, and the fibers containing TPZ (8–95 mg g^−1^) were called TPZ@F.

### Fabrication and Characterization of Sandwich‐Structured Composite Composed of Electrospun Fibers and 3D‐Printed Scaffolds—Characterizations of Electrospun Fibers

To observe the microtopography and determine the microstructural characteristics of prepared electrospun fibers, TPZ@F was characterized with a SEM (Quanta 400F, Oxford, UK) and a fluorescence microscope (Ni‐U, Nikon, Japan). Before capturing the SEM images of fibers, an ion sputter coater (MC1000, Hitachi, Japan) was used to coat the samples with gold for 3 min in vacuo. The diameter of each fiber in the acquired SEM images was measured using the software Fiji (Version 1.53q, USA). As for the observation with fluorescence, First the absorption spectrum of TPZ in an aqueous solution was detected and an absorption peak at around 470 nm was found. Then, an excitation filter labeled with FITC to observe the TPZ@F and TPZ@F‐integrated patches was used.

### Fabrication and Characterization of Sandwich‐Structured Composite Composed of Electrospun Fibers and 3D‐Printed Scaffolds—Rheological Analyses of 3D‐Printing Ink

The rheological behaviors characterized by viscoelastic properties were investigated using a HAAKE Rheometer (MARS 60, Thermo, USA) carrying a parallel plate (diameter = 27.83 mm) on a homeothermic platform at a constant temperature of 25 °C. The test distance between the plate and sample‐loading platform was 1.0 mm. During the shear strain sweep process, the oscillatory frequency was fixed at 1.0 Hz, and each sample's *G*' and *G*" moduli were recorded with the strain varying from 0.2% to 100.0%. The 3D‐printing ink formula based on the strain sweep results was preliminarily acquired. To further confirm the feasibility of the ink preparation approach, a shear thixotropy test at a constant frequency of 1 Hz and three‐stage strains (0.8–80–0.8%) with each stage duration of 3 min was carried out.

### Fabrication and Characterization of Sandwich‐Structured Composite Composed of Electrospun Fibers and 3D‐Printed Scaffolds—Fabrication of 3D‐Printed Scaffolds and Sandwich‐Structured Composites

The scaffolds were 3D‐printed according to the method in the previous report.^[^ ^6c^ ^]^ In the beginning, SPI (10%, w/v) was dispersed in ultrapure water via stirring for 2 h at room temperature, followed by the addition of PVA (10%, w/v) and stirring for another 2 h. The uniform dispersion was then heated and stirred for 30 min in a water bath at 95 °C. After cooling for 1 h at 4 °C, to produce the 3D printing ink, CaCl_2_ (0.2% w/v), SA (5.25%, w/v), and CA4P at different concentrations were added and fully mixed with the dispersion. Before printing, the suitable shape and printing parameters were designed with the software Simplify3D (Version 4.1, USA). Subsequently, the scaffolds were printed at a 2.0–3.0 kg cm^−2^ compressed air pressure and a 33% v/v filling rate. During the printing, one fiber patch was sandwiched between two 3D‐printed scaffolds to construct a scaffold‐fiber‐scaffold (S‐F‐S) composite. In the end, the composites were prefrozen for 2 h at −80 °C and lyophilized overnight. The scaffold without CA4P was called blank S, the scaffold containing CA4P (0.4–6.0 mg g^−1^) was named CA4P@S, the S‐F‐S composite without TPZ or CA4P was called blank S‐F‐S, the S‐F‐S composite only containing TPZ in fibers was named S‐TPZ@F‐S, the S‐F‐S composite only containing CA4P in scaffolds (0.4–6.0 mg g^−1^) was called CA4P@S‐F‐CA4P@S. The S‐F‐S composite containing TPZ in fibers and CA4P in scaffolds was called CA4P@S‐TPZ@F‐CA4P@S (only used in the animal experiments), of which both TPZ‐ and CA4P‐loading amounts were 1.7 mg g^−1^ in the whole sandwich‐like implant.

### Fabrication and Characterization of Sandwich‐Structured Composite Composed of Electrospun Fibers and 3D‐Printed Scaffolds—Characterizations of Scaffolds and Sandwich‐Structured Composites

The visual appearances of fabricated scaffolds and sandwich‐structured composites were observed and photographed with a digital camera (RX100M3, Sony, Japan), and the microstructures were observed and evaluated based on the SEM images obtained by the methods mentioned above. To estimate the containing or loading of used materials or drugs in scaffolds, a typical element mapping was conducted with an SEM equipped with an EDS (INCA Energy, Oxford, UK), and the EDS mapping graphs were acquired.

### Fabrication and Characterization of Sandwich‐Structured Composite Composed of Electrospun Fibers and 3D‐Printed Scaffolds—Drug Release In Vitro

To mimic and assess the in vivo release of TPZ and CA4P as closely as possible, three forms of drug‐loaded composites, including CA4P@S, TPZ@F, and S‐TPZ@F‐S, were placed in dialysis tubes with a cut‐off molecular weight of 3500 Da, immersed in 15 mL PBS (10 mm) at pH 7.4 or pH 6.5, and slowly oscillated (50 rpm) at 37 °C. During the release monitoring, 200 µL released solution was collected at the predetermined time point, and 200 µL fresh PBS was added to maintain the constant volume of the release system. Notably, RhB, with the similar solubility to CA4P, was loaded into scaffolds to mimic CA4P release. The concentration of TPZ or RhB in the released solution was determined based on the absorbance value at 470 or 554 nm and the associated standard curve. After continuous measuring for 14 d with a microplate reader (Synergy H1MF, BioTek, US), the cumulative release of TPZ or RhB was calculated, and the corresponding release profiles were plotted.

### Fabrication and Characterization of Sandwich‐Structured Composite Composed of Electrospun Fibers and 3D‐Printed Scaffolds—Degradation In Vitro

To evaluate the degradability of used composites, all the blank scaffolds or electrospun fibers were first prepared with the same model specifications and immersed in PBS at pH 7.4 or 6.5. Then, the blank scaffolds and fibers in PBS with or without 8 µg mL^−1^ proteinase K and PBS with or without 20 U mL^−1^ lipase, respectively, were incubated for 12 d at 37 °C. During the degradation, the digital photographs of samples in PBS were captured at the predetermined time points. After every photographing session, three scaffolds were taken from PBS, frozen in liquid nitrogen, and lyophilized to acquire the SEM images. In the end, all the scaffolds were lyophilized and weighed, and the weight losses were calculated.

### Cell Culture

A murine prostate cancer cell line RM‐1 was purchased from the National Collection of Authenticated Cell Cultures (Shanghai, China), and the HUVECs were obtained from iCell Bioscience (Shanghai, China). RM‐1 cells were cultured in basic Dulbecco's modified eagle medium (DMEM, Gibco, USA) containing high‐content glucose (4.5 g L^−1^) and 1 mm sodium pyruvate, supplemented by 10% v/v fetal bovine serum (FBS, Biological Industries, Israel) and 1% w/v penicillin/streptomycin (P/S). HUVECs were cultured in DMEM/Ham's F‐12 (1:1 mixed) with 2.5 mm L‐glutamine and 15 mm 2‐[4‐(2‐Hydroxyethyl)‐1‐piperazinyl]‐ethanesulfonic acid (DMEM/F‐12, Corning, USA), supplemented by 10% FBS and 1% P/S. Normally, RM‐1 cells and HUVECs were incubated at 37 °C in a humidified atmosphere of 5% CO_2_ and 95% air with an O_2_ concentration of about 21%. A hypoxic condition for incubating RM‐1 cells was created in a small tris‐gas incubator (Galaxy 48 R, Eppendorf, Germany) under 5% CO_2_, 94% N_2_, and only 1% O_2_. Both the hypoxic extracellular and intracellular microenvironments were achieved by preincubating cells in the hypoxic incubator for at least 12 h.

### Colony Formation Assay

The colony formation ability of HUVECs was assessed according to the methods described in previous reports with a few modifications.^[^ ^37^ ^]^ After seeding in 6‐well plates (2 × 10^2^ cells per well) and preincubation for 48 h, HUVECs were treated with scaffolds containing CA4P at different concentrations, followed by a prolonged coincubation for 7 d. Then, the scaffolds and transwell permeable supports were removed, and the formed cell colonies were washed 3 times with prewarmed PBS. Before the staining with 0.5% w/v crystal violet, the washed cell colonies were fixed with the 4% w/v PFA (dissolved in PBS) solution. At last, the pictures of stained cell colonies were taken by a digital camera, and the colony number in each well was counted with Fiji.

### Investigation of Inhibitory and Destructive Effect of CA4P@S on Blood Vessels In Vitro—Suppressive effect of CA4P@S on the Migration of HUVECs

The migration ability of HUVECs was estimated using the classic scratch wound healing assay.^[^ ^38^ ^]^ To begin, HUVECs were seeded in 6‐well plates (5 × 10^5^ cells per well) and preincubated until the bottom of each well was full of adherent cells. Then, the scratches were created with 200 µL pipette tips moving along a ruler. After the coincubation with CA4P@S containing CA4P at various concentrations for 24 or 48 h, the HUVEC brightfield images indicating wound healing were captured with an inverted fluorescence microscope and semiquantitatively analyzed using Fiji.

### Investigation of Inhibitory and Destructive Effect of CA4P@S on Blood Vessels In Vitro—Capillary Tube Formation Assay

According to the methods demonstrated in previous studies, the capillary tube formation assay was performed to evaluate the impact of CA4P@S on blood vessels in vitro. Before seeding of HUVECs, 200 µL Matrigel (BD Biosciences, USA) was pipetted and spread evenly in every well of 24‐well plates without causing bubbles, and then allowed to solidify for 30 min at 37 °C. HUVECs were seeded in the 24‐well plates (8 × 10^4^ cells per well) with formed gels to investigate the inhibition of angiogenesis. At the same time, FGF2 (5 ng mL^−1^) and different CA4P@S composites were simultaneously added, followed by the coincubation for 6 h. Subsequently, HUVECs were stained with calcein‐AM for 30 min at 37 °C, and the washed cells were observed with an inverted fluorescence microscope. To assess the damage to preexisting blood vessels, various CA4P@S composites were added after the establishment of capillary networks formed by HUVECs stimulated with FGF2 for 6 h at 37 °C. Then the images of HUVECs at predetermined time points were captured, and calcein‐AM was used to stain the live cells after coincubation for 12 h. Finally, the image quantification analyses of these two experiments were performed using Fiji.

### Investigation of Cytotoxicity of TPZ in Different States In Vitro—ΔΨm Assay

Overaccumulated ROS can disrupt mitochondria and reduce ΔΨm. Besides, the protonated amidogens on TPZ and its reduction product BTZ can also be electrostatically attracted to the negatively charged mitochondrial membrane through electrostatic attractions, thus decreasing ΔΨm, and further inducing mitochondrial dysfunction.^[^ ^25^ ^]^ Initially, RM‐1 cells were seeded in 6‐well plates (3 × 10^5^ cells per well) and preincubated for 12 h. Adherently growing RM‐1 cells were then incubated in normoxia or hypoxia for 12 h, and then coincubated with TPZ in different states under the same conditions for another 12 h. Afterward, the washed RM‐1 cells were stained with 10 µm JC‐1 for 30 min at 37 °C in dark environments. Before flow cytometry, the washed RM‐1 cells were digested with trypsin, fixed with PFA, and washed with PBS again. Before the observation with an inverted fluorescence microscope, the fixed and washed RM‐1 cells were stained with DAPI (1 µg mL^−1^) for 15 min at room temperature.

### Investigation of Cytotoxicity of TPZ in Different States In Vitro—Intracellular ATP Detection

Following the adherent growth in 6‐well plates (3 × 10^5^ cells per well) and precultured in the atmosphere with various O_2_ concentrations, the RM‐1 cells were cocultured with different forms of TPZ for 24 h. Afterward, the cells were washed 3 times with PBS precooled at 4 °C and lysed with the precooled lysis buffer (Beyotime) solution on ice. The cell lysates were centrifugated (12 000 g) for 15 min at 4 °C, and the supernatants were collected. The protein concentration and ATP level in the supernatant of each sample were determined using a bicinchoninic acid (BCA) protein assay kit (Dingguo, Beijing, China) and an ATP detection kit (Beyotime) according to the corresponding manufacturer's protocols, respectively.

### Inhibition of Tumor Postsurgical Recurrence and Metastasis In Vivo—Animal Model

All animal experiments and handling procedures were approved by Institutional Animal Care and Use Committee, Sun Yat‐Sen University (SYSU‐IACUC‐2022‐001812). First, an experimental prostate cancer model was established via subcutaneously injecting 2 × 10^6^ RM‐1 cells (suspended in 100 µL PBS) into the right flank of the lower back in each male C57BL/6 mouse with the age of 6–8 weeks (GemPharmatech, Guangzhou, China). The tumor volume (V) was calculated based on the following equation: V = (tumor length) × (tumor width)^2^/2.^[^ ^39^ ^]^ In the following days, the lengths and widths of tumors were measured daily using a vernier caliper until the volumes reached about 450 mm^3^ on day 7 post tumor inoculation. Then, to simulate positive margins in clinical operations, 90% of histologically confirmed tumor tissue was resected from each tumor‐bearing mouse after the anesthetization by intraperitoneal injection of the sterilized 0.6% w/v sodium pentobarbital solution (100 µL g^−1^).

### Inhibition of Tumor Postsurgical Recurrence and Metastasis In Vivo—Implantation and Therapy

Immediately following the excision, different composites were implanted at the sites of tumor residues. Given the compositions of implants, the mice were divided into 6 groups: control, blank S‐F‐S, free TPZ (5 µg g^−1^) + CA4P (5 µg g^−1^), S‐TPZ@F‐S (TPZ, 5 µg g^−1^), CA4P@S‐ F‐CA4P@S (CA4P, 5 µg g^−1^), and CA4P@S‐TPZ@F‐CA4P@S (CA4P, 5 µg g^−1^; TPZ, 5 µg g^−1^). After implantation, the incisions were sutured, and each mouse received the subcutaneous injection with the 0.5% w/v antibiotic solution (500 µL) to prevent infection. Notably, all the surgical instruments and implants were sterilized by autoclaving, ultraviolet irradiation, or 75% v/v ethanol spraying, and all surgical procedures were performed on temperature‐keeping pads in a sterile environment. After surgery, the mice's tumor volumes and body weights were measured and recorded every 2 days during the treatment period of 10 d.

### Inhibition of Tumor Postsurgical Recurrence and Metastasis In Vivo—Transcriptome Sequencing and Analysis

First, about 100 mg of frozen tumor tissues from one mouse were thoroughly cryoground in 1 mL Trizol regent (Beyotime) with a refrigerated tissue homogenizer (KZ‐III‐FP, Servicebio). Subsequently, the total RNA of tumor tissues was extracted according to the manufacturer's instructions of the Trizol regent, and the genomic DNA was eliminated using DNase I (Beyotime). After that, the RNA concentration and quality of isolated samples were determined with a Nanodrop One spectrophotometer (Thermo) and a Bioanalyzer 2100 (Agilent, USA), and only high‐quality RNA samples (OD_260/280_ = 1.8–2.2, OD_260/230_ ≥ 2.0, and RIN > 7.0) were used to construct the sequencing library.^[^ ^40^ ^]^ Then the mRNA purification, fragmentation, reverse transcription, polymerase chain reaction, library construction, and sequencing using the Novaseq 6000 system (Illumina, USA) were implemented at LC‐Bio Technology (Hangzhou, China). The raw reads might contain adapters, and low‐quality or repeated bases, which affected the following assembly and analysis. Thus, to get high‐quality clean reads, the obtained reads were further filtered using the software Cutadapt (https://cutadapt.readthedocs.io/en/stable/, Version 4.1). Then the clean reads of each sample were separately aligned to the reference genome (http://ftp.ensembl.org/pub/release‐92/fasta/mus_musculus_c57bl6nj/dna/) using the software HISAT (https://daehwankimlab.github.io/hisat2/, Version 2.2.1).^[^ ^41^ ^]^ After the orientated mapping, the reads were assembled using the software StringTie (http://ccb.jhu.edu/software/stringtie/,

Version 2.2.0) with default parameters, and the transcriptomes of all samples were merged to reconstruct a comprehensive transcriptome using the software GffCompare (http://ccb.jhu.edu/software/stringtie/gffcompare.shtml, Version 0.12.6).^[^ ^41^, ^42^ ^]^

Following the generation of the final transcriptome, the software Ballgown (http://www.bioconductor.org/packages/release/bioc/html/ballgown.html, Version 2.28.0) and StringTie were used to assess the expression levels of transcripts and the expression abundance of mRNAs by calculating the fragment per kilobase of transcript per million mapped reads (FPKM) value.^[^ ^41^, ^42^ ^]^ Differentially expressed genes (DEGs) were then analyzed with the software DESeq2 (https://www.home‐for‐researchers.com/paper/index.html#/, Version 1.36.0) between the two groups of control and CA4P@S‐TPZ@F‐CA4P@S, and identified as the genes with absolute FC > 2, and *P* value or false discovery rate < 0.05, and then subjected to the following enrichment analysis.^[^ ^40^, ^43^ ^]^ Finally, the plots of UpSet and volcano, enrichment analyses of KEGG pathways and GO terms, and GSEA were graphed and analyzed on the cloud‐based analysis platform (https://www.omicstudio.cn/tool) supported by LC‐Bio Technology, the heatmaps were plotted with the software TBtools (https://github.com/CJ‐Chen/TBtools/releases, Version 1.098761), and the circled enrichment maps were graphed using the online tool Sangerbox 3.0 (http://vip.sangerbox.com/home.html). In addition, the protein–protein interactions of genes were analyzed and plotted with the online tool STRING (http://www.string‐db.org/, Version 11.5) and the software Cytoscape (https://cytoscape.org/, Version 3.9.0).^[^ ^44^ ^]^

### Statistics

Origin (version 2021) was used to graph the data, and GraphPad Prism (version 9.0) was used for the statistical analyses. All data are presented as mean ± SD, and the SD values are indicated by error bars. Unless otherwise stated, the mean and SD were calculated based on three or more replicates. One‐way ANOVA was performed to estimate the significance of differences among three or more groups. Two‐way ANOVA was carried out in the data comparison among three or more groups with two independent nominal‐level variables. Asterisks and pounds with various numbers indicate significant inter‐group and intra‐group differences at distinct levels (* or # *P* < 0.05, ** or ## *P* < 0.01, and *** or ### *P* < 0.001), respectively. n.s. represents no significance.

## Conflict of Interest

The authors declare no conflict of interest.

## Supporting information

Supporting Information

## Data Availability

The data that support the findings of this study are available from the corresponding author upon reasonable request.

## References

[1] R. L. Siegel , K. D. Miller , H. E. Fuchs , A. Jemal , Ca‐Cancer J. Clin. 2022, 72, 7. 35020204 10.3322/caac.21708

[2] a) T. Todo , H. Ito , Y. Ino , H. Ohtsu , Y. Ota , J. Shibahara , M. Tanaka , Nat. Med. 2022, 28, 1630; 35864254 10.1038/s41591-022-01897-x PMC9388376

[3] a) G. Q. Wei , Y. Wang , G. Yang , Y. Wang , R. Ju , Theranostics 2021, 11, 6370; 33995663 10.7150/thno.57828 PMC8120226

[4] Y. Ding , Z. Tong , L. Jin , B. Ye , J. Zhou , Z. Sun , H. Yang , L. Hong , F. Huang , W. Wang , Z. Mao , Adv. Mater. 2022, 34, 2106388. 10.1002/adma.202106388 34821416

[5] a) X. Zhou , Z. Yao , H. Bai , J. Duan , Z. Wang , X. Wang , X. Zhang , J. Xu , K. Fei , Z. Zhang , F. Tan , Q. Xue , S. Gao , Y. Gao , J. Wang , J. He , Lancet Oncol. 2021, 22, 1265; 34391508 10.1016/S1470-2045(21)00333-8

[6] a) Y. Hu , Y. Xu , R. L. Mintz , X. Luo , Y. Fang , Y.‐H. Lao , H. F. Chan , K. Li , S. Lv , G. Chen , Y. Tao , Y. Luo , M. Li , Biomaterials 2023, 293, 121942; 36512863 10.1016/j.biomaterials.2022.121942

[7] a) K. Guo , X. Ma , J. Li , C. Zhang , L. Wu , Eur. J. Med. Chem. 2022, 241, 114660; 35964428 10.1016/j.ejmech.2022.114660

[8] a) M. Zweifel , G. C. Jayson , N. S. Reed , R. Osborne , B. Hassan , J. Ledermann , G. Shreeves , L. Poupard , S. P. Lu , J. Balkissoon , D. J. Chaplin , G. J. S. Rustin , Ann. Oncol. 2011, 22, 2036; 21273348 10.1093/annonc/mdq708

[9] a) Z. Ma , X. Xiang , S. Li , P. Xie , Q. Gong , B.‐C. Goh , L. Wang , Semin. Cancer Biol. 2022, 80, 379; 33002608 10.1016/j.semcancer.2020.09.011

[10] J. Griggs , J. C. Metcalfe , R. Hesketh , Lancet Oncol. 2001, 2, 82. 11905799 10.1016/S1470-2045(00)00224-2

[11] a) K. Zhang , L. Zhu , Y. Zhong , L. Xu , C. Lang , J. Chen , F. Yan , J. Li , J. Qiu , Y. Chen , D. Sun , G. Wang , K. Qu , X. Qin , W. Wu , Adv. Sci. 2023, 10, 2205422; 10.1002/advs.202205422 PMC9896077 36507607

[12] W. R. Wilson , M. P. Hay , Nat. Rev. Cancer 2011, 11, 393. 21606941 10.1038/nrc3064

[13] a) L. Zhang , X. Du , Q. Li , L. Qian , J. Chen , C. Liu , Q. Yu , Z. Gan , Adv. Funct. Mater. 2022, 32, 2204629;

[14] a) B. Hong , V. W. Y. Lui , E. P. Hui , M. H. L. Ng , S.‐H. Cheng , F. L. Sung , C.‐M. Tsang , S.‐W. Tsao , A. T.‐C. Chan , Invest. New Drugs 2011, 29, 401; 20013349 10.1007/s10637-009-9356-z

[15] a) B. Yang , C. P. Reynolds , Clin. Cancer Res. 2005, 11, 2774; 15814660 10.1158/1078-0432.CCR-04-2382

[16] S. Yang , Z. Tang , C. Hu , D. Zhang , N. Shen , H. Yu , X. Chen , Adv. Mater. 2019, 31, 1805955. 10.1002/adma.201805955 30680816

[17] Y.‐T. Xu , L.‐l. Liu , J. Agric. Food Chem. 2016, 64, 7275. 27608266 10.1021/acs.jafc.6b02737

[18] A. Mohammed , A. Elshaer , P. Sareh , M. Elsayed , H. Hassanin , Int. J. Pharm. 2020, 580, 119245. 32201252 10.1016/j.ijpharm.2020.119245

[19] J. Chen , Z. Jiang , W. Xu , T. Sun , X. Zhuang , J. Ding , X. Chen , Nano Lett. 2020, 20, 6191. 32697585 10.1021/acs.nanolett.0c02515

[20] C. Deng , J. Zhao , S. Zhou , J. Dong , J. Cao , J. Gao , Y. Bai , H. Deng , Mol. Ther. 2020, 28, 75. 31672285 10.1016/j.ymthe.2019.10.010 PMC6953963

[21] S. M. Nabha , R. M. Mohammad , M. H. Dandashi , B. Coupaye‐Gerard , A. Aboukameel , G. R. Pettit , A. M. Al‐Katib , Clin. Cancer Res. 2002, 8, 2735. 12171907

[22] a) C. Dumontet , M. A. Jordan , Nat. Rev. Drug Discovery 2010, 9, 790; 20885410 10.1038/nrd3253 PMC3194401

[23] P. Nowak‐Sliwinska , K. Alitalo , E. Allen , A. Anisimov , A. C. Aplin , R. Auerbach , H. G. Augustin , D. O. Bates , J. R. van Beijnum , R. H. F. Bender , G. Bergers , A. Bikfalvi , J. Bischoff , B. C. Böck , P. C. Brooks , F. Bussolino , B. Cakir , P. Carmeliet , D. Castranova , A. M. Cimpean , O. Cleaver , G. Coukos , G. E. Davis , M. De Palma , A. Dimberg , R. P. M. Dings , V. Djonov , A. C. Dudley , N. P. Dufton , S.‐M. Fendt , et al., Angiogenesis 2018, 21, 425.29766399

[24] a) L. Vincent , P. Kermani , L. M. Young , J. Cheng , F. Zhang , K. Shido , G. Lam , H. Bompais‐Vincent , Z. Zhu , D. J. Hicklin , P. Bohlen , D. J. Chaplin , C. May , S. Rafii , J. Clin. Invest. 2005, 115, 2992; 16224539 10.1172/JCI24586 PMC1253622

[25] J. Qin , N. Gong , Z. Liao , S. Zhang , P. Timashev , S. Huo , X.‐J. Liang , Nanoscale 2021, 13, 7108. 33889907 10.1039/d1nr01068a

[26] B. G. Siim , P. L. van Zijl , J. M. Brown , Br. J. Cancer 1996, 73, 952. 8611431 10.1038/bjc.1996.187 PMC2075810

[27] D. Shi , H. Zhang , H. Zhang , L. Li , S. Li , Y. Zhao , C. Zheng , G. Nie , X. Yang , Chem. Eng. J. 2022, 433, 134357.

[28] a) J. Bariwal , H. Ma , G. A. Altenberg , H. Liang , Chem. Soc. Rev. 2022, 51, 1702; 35156110 10.1039/d1cs01074c

[29] a) M. Ishikawa , M. Hosokawa , N. Oh‐hara , Y. Niho , H. Kobayashi , J. Natl. Cancer Inst. 1987, 78, 567; 3469467

[30] a) V. Cortez‐Retamozo , M. Etzrodt , A. Newton , P. J. Rauch , A. Chudnovskiy , C. Berger , R. J. H. Ryan , Y. Iwamoto , B. Marinelli , R. Gorbatov , R. Forghani , T. I. Novobrantseva , V. Koteliansky , J.‐L. Figueiredo , J. W. Chen , D. G. Anderson , M. Nahrendorf , F. K. Swirski , R. Weissleder , M. J. Pittet , Proc. Natl. Acad. Sci. USA 2012, 109, 2491; 22308361 10.1073/pnas.1113744109 PMC3289379

[31] a) S. E. A. Street , E. Cretney , M. J. Smyth , Blood 2001, 97, 192; 11133760 10.1182/blood.v97.1.192

[32] B. Zhao , Z. Dong , W. Liu , F. Lou , Q. Wang , H. Hong , Y. Wang , J. Nanobiotechnol. 2021, 19, 124. 10.1186/s12951-021-00865-w PMC8088584 33933077

[33] a) L. O. C. L. van Kempen , L. M. Coussens , Cancer Cell 2002, 2, 251; 12398887 10.1016/s1535-6108(02)00157-5

[34] a) S. Io , M. Kabata , Y. Iemura , K. Semi , N. Morone , A. Minagawa , B. Wang , I. Okamoto , T. Nakamura , Y. Kojima , C. Iwatani , H. Tsuchiya , B. Kaswandy , E. Kondoh , S. Kaneko , K. Woltjen , M. Saitou , T. Yamamoto , M. Mandai , Y. Takashima , Cell Stem Cell 2021, 28, 1023; 33831365 10.1016/j.stem.2021.03.013

[35] a) X. Sun , Y. Zhang , J. Li , K. S. Park , K. Han , X. Zhou , Y. Xu , J. Nam , J. Xu , X. Shi , L. Wei , Y. L. Lei , J. J. Moon , Nat. Nanotechnol. 2021, 16, 1260; 34594005 10.1038/s41565-021-00962-9 PMC8595610

[36] S. L. Chan , M. Schuler , Y.‐K. Kang , C.‐J. Yen , J. Edeline , S. P. Choo , C.‐C. Lin , T. Okusaka , K.‐H. Weiss , T. Macarulla , S. Cattan , J.‐F. Blanc , K.‐H. Lee , M. Maur , S. Pant , M. Kudo , E. Assenat , A. X. Zhu , T. Yau , H. Y. Lim , J. Bruix , A. Geier , C. Guillen‐Ponce , A. Fasolo , R. S. Finn , J. Fan , A. Vogel , S. Qin , M. Riester , V. Katsanou , et al., J. Exp. Clin. Cancer Res. 2022, 41, 189. 35655320 10.1186/s13046-022-02383-5 PMC9161616

[37] a) J. Guo , J. Cheng , N. Zheng , X. Zhang , X. Dai , L. Zhang , C. Hu , X. Wu , Q. Jiang , D. Wu , H. Okada , P. P. Pandolfi , W. Wei , Adv. Sci. 2021, 8, 2004303; 10.1002/advs.202004303 PMC8456201 34278744

[38] C. Xian , Z. Zhang , X. You , Y. Fang , J. Wu , Adv. Funct. Mater. 2022, 32, 2202410.

[39] a) S. Lei , J. Zhang , N. T. Blum , M. Li , D.‐Y. Zhang , W. Yin , F. Zhao , J. Lin , P. Huang , Nat. Commun. 2022, 13, 1298; 35277519 10.1038/s41467-022-29082-1 PMC8917194

[40] T. Liu , B. Xiao , F. Xiang , J. Tan , Z. Chen , X. Zhang , C. Wu , Z. Mao , G. Luo , X. Chen , J. Deng , Nat. Commun. 2020, 11, 2788. 32493916 10.1038/s41467-020-16544-7 PMC7270130

[41] a) D. Kim , J. M. Paggi , C. Park , C. Bennett , S. L. Salzberg , Nat. Biotechnol. 2019, 37, 907; 31375807 10.1038/s41587-019-0201-4 PMC7605509

[42] a) S. Kovaka , A. V. Zimin , G. M. Pertea , R. Razaghi , S. L. Salzberg , M. Pertea , Genome Biol. 2019, 20, 278; 31842956 10.1186/s13059-019-1910-1 PMC6912988

[43] a) S. M. E. Sahraeian , M. Mohiyuddin , R. Sebra , H. Tilgner , P. T. Afshar , K. F. Au , N. Bani Asadi , M. B. Gerstein , W. H. Wong , M. P. Snyder , E. Schadt , H. Y. K. Lam , Nat. Commun. 2017, 8, 59; 28680106 10.1038/s41467-017-00050-4 PMC5498581

[44] a) N. Kong , R. Zhang , G. Wu , X. Sui , J. Wang , N. Y. Kim , S. Blake , D. De , T. Xie , Y. Cao , W. Tao , Proc. Natl. Acad. Sci. USA 2022, 119, e2112696119; 35131941 10.1073/pnas.2112696119 PMC8851555

