# Supplementary material for: Sandwich‐Structured Implants to Obstruct Multipath Energy Supply and Trigger Self‐Enhanced Hypoxia‐Initiated Chemotherapy Against Postsurgical Tumor Recurrence and Metastasis
Source: Adv Sci (Weinh). 2023 May 8;10(22):2300899. doi: 10.1002/advs.202300899 (PMC10401165; doi:10.1002/advs.202300899)
Supplement: Supplementary file 1 — Supporting Information [file ADVS-10-2300899-s001.pdf]

ADVANCED SCIENCE
Open Access

Supporting Information

for *Adv. Sci.*, DOI 10.1002/advs.202300899

Sandwich-Structured Implants to Obstruct Multipath Energy Supply and Trigger Self-Enhanced Hypoxia-Initiated Chemotherapy Against Postsurgical Tumor Recurrence and Metastasis

Youqiang Fang, Xing Luo, Yanteng Xu, Zheng Liu, Rachel L. Mintz, Haiyang Yu, Xuan Yu, Kai Li, Enguo Ju, Haixia Wang, Zhaohui Tang*, Yu Tao* and Mingqiang Li**

Supporting Information

Sandwich-Structured Implants to Obstruct Multipath Energy Supply and Trigger Self-Enhanced Hypoxia-Initiated Chemotherapy Against Postsurgical Tumor Recurrence and Metastasis

Youqiang Fang, Xing Luo, Yanteng Xu, Zheng Liu, Rachel L. Mintz, Haiyang Yu, Xuan Yu, Kai Li, Enguo Ju, Haixia Wang, Zhaohui Tang,* Yu Tao,* and Mingqiang Li**

Y. Fang, X. Luo, Y. Xu, Z. Liu, X. Yu, K. Li, E. Ju, H. Wang, Y. Tao, M. Li
Laboratory of Biomaterials and Translational Medicine
Center for Nanomedicine
Department of Urology
The Third Affiliated Hospital
Sun Yat-sen University
Guangzhou 510630, China
E-mail: xuyt59@mail.sysu.edu.cn; taoy28@mail.sysu.edu.cn; limq567@mail.sysu.edu.cn

R. L. Mintz
Department of Biomedical Engineering
Washington University in St. Louis
St. Louis, MO, 63110, USA

H. Yu, Z. Tang
Key Laboratory of Polymer Ecomaterials
Changchun Institute of Applied Chemistry
Chinese Academy of Sciences
Changchun 130022, P. R. China
E-mail: ztang@ciac.ac.cn

X. Yu, K. Li
Department of Ultrasound
The Third Affiliated Hospital
Sun Yat-sen University
Guangzhou 510630, China

Y. Tao, M. Li
Guangdong Provincial Key Laboratory of Liver Disease Research
Guangzhou 510630, China

Experimental Section

Measurement of Cell Viability

Cell viability was evaluated using the CCK8 assay. Firstly, cells were seeded in 24-well plates (8×10^4 cells per well) and pre-incubated for 12 h. Subsequently, different concentrations of CA4P under free state or in the scaffolds were added into each well of HUVEC-seeded plates. Various concentrations of TPZ under free state, in the electrospun fibers or the S-F-S composites, were added into each well of RM-1 cell-seeded plates. Of note, transwell permeable supports (Corning) were used in the wells with fibers, scaffolds, or sandwich-structured composites, avoiding the cell death caused by the sedimentations of fragmentized carriers. After co-incubation for 12 h, 24 h, or 48 h, the transwell permeable supports were removed, the cells were washed 3 times with PBS (10 mM, pH 7.2–7.4) pre-warmed at 37 °C, and the cell viabilities were determined using CCK8 kits (Beyotime).

Live/Dead Staining

Following pre-incubation for 12 h in 24-well plates (8×10^4 cells per well), HUVECs and RM-1 cells were co-incubated with CA4P and TPZ under free state or in the carriers, respectively. After that, the cells were stained simultaneously with 2.5 μM calcein-AM (green fluorescence referring to live cells) and 1.5 μM PI (red fluorescence indicating dead cells) for 30 min at 37 °C. Finally, the fluorescence images were captured with an inverted fluorescence microscope (Ti2-U, Nikon, Japan).

CA4P@S-Caused Inhibition of Tubulin Aggregation in HUVECs

It has been reported that CA4P can inhibit tubulin aggregation during mitosis, thus preventing cells from progressing through the G2/M phase, and further compromising their growth and proliferation.[1] To determine the suppressive effect of CA4P@S on vascular endothelial cells in vitro, HUEVCs were seeded in 24-well plates (8×10^4 cells per well) with one polylysine-coated glass coverslip in every well and pre-incubated for 12 h to achieve their adherent growth on the coverslips. Following the co-incubation with CA4P@S containing CA4P at different concentrations for 12 h, HUEVCs were washed with pre-warmed PBS and stained with a tubulin-tracker green staining kit (Beyotime) for 30 min in the dark. After washing with PBS and fixation with a PFA solution, each coverslip was gently taken out and shifted onto a glass slide carrying one drop of antifade mounting medium with DAPI (Beyotime). In the end, the fluorescence images were acquired using a CLSM (TCS SP8, Leica), and the fluorescence analysis was conducted using Fiji.

Effect of CA4P@S on Cell Cycle of HUVECs

Initially, HUVECs were seeded in 6-well plates (3 × 10^5 cells per well) and pre-incubated for 12 h, followed by the co-incubation with CA4P@S containing CA4P at various concentrations for another 12 h. Subsequently, the washed HUVECs were digested with 0.25% (w/v) trypsin (Beyotime) and washed by 3 cycles of centrifugation (300 g) for 5 min and complete resuspension in PBS. Following the fixation with 75% (v/v) ethanol, the suspended HUVECs were washed 3 times with PBS and treated with RNase (0.25 mg mL^{-1}) for 30 min at 37 ºC. After that, the washed HUVECs were stained with PI (5 μg mL^{-1}) for 30 min in the dark. Finally, the fluorescence analysis of HUVECs was performed with a flow cytometer (CytoFLEX S, Beckman, USA).

Intracellular ROS Detection

Previous reports have also demonstrated that ROS can be generated during the bioreduction of TPZ, and the reduced BTZ also can release free radicals. Additionally, the dysfunctional mitochondria can produce and accumulate ROS, further enhancing the destruction of mitochondria.[2] After seeding in 6-well plates (3 × 10^5 cells per well) and pre-incubation for adherent growth, RM-1 cells were transferred into normoxic or hypoxic environments and incubated for 12 h, followed by the co-incubation with different composites with or without TPZ for another 12 h. To detect the intracellular ROS level, the washed RM-1 cells were stained with DCFH-DA (10 μM) for 30 min in the dark and sequentially fixed with PFA. The suspended RM-1 cells were analyzed with a flow cytometer, and the adherent RM-1 cells were observed using an inverted fluorescence microscope following the staining with DAPI.

Flow Cytometry of Cell Apoptosis

After the adherent growth in 6-well plates (3 × 10^5 cells per well) under normoxic conditions, the RM-1 cells were cultured in normoxia or hypoxia for 12 h, followed by the co-incubation with TPZ in various states for another 12 h. Subsequently, the treated RM-1 cells were gently washed with PBS, moderately digested with trypsin without EDTA, and stained with an Annexin V-FITC/PI dual-staining kit (KeyGEN, China) for 30 min under dark conditions. The stained and washed RM-1 cells were analyzed by flow cytometry.

Western Blotting

Following pre-incubation in 6-well plates (3 × 10^5 cells per well), the adherently growing RM-1 cells were incubated under normoxic or hypoxic conditions for 12 h. Then the RM-1 cells

were co-incubated with TPZ in different states for another 12 h, followed by washing with pre-cooled PBS. After that, the cells were lysed with the pre-cooled radio-immunoprecipitation assay (RIPA) buffer solution containing 1 mM phenylmethanesulfonyl fluoride (a protease inhibitor, Beyotime) on ice. The collected cell lysates were centrifugated (12000 g) for 15 min at 4 °C. The protein concentrations of supernatants were measured using a BCA protein assay kit and equalized through the dilution with the RIPA buffer solution. All the harvested samples were mixed with the 5 × reducing sample loading buffer solution (Beyotime) at a 1 : 4 v/v ratio and denatured through heating (95 °C) and oscillation (1200 rpm) for 30 min in a shaking incubator. For each sample, 40 μg total protein was used in the SDS-PAGE, followed by the protein transference from a gel to a nitrocellulose (NC) membrane with a pore size of 0.22 μm at 4 °C. Subsequently, the NC membranes were blocked with 5% (w/v) skim milk containing antibacterial agents for 2 h at room temperature. Various rabbit-derived primary antibodies against β-actin (AF5003, Beyotime), MMP9 (AF5234, Beyotime), HIF1α (AG2135, Beyotime), and VEGF (AF0312, Beyotime) were diluted and co-incubated with the washed NC membranes overnight at 4 °C. The next day, the washed membranes were co-incubated with the HRP-conjugated goat-derived immunoglobulin G (IgG) against rabbit (secondary antibody, A0208, Beyotime) for 1 h at room temperature. Following the last washing with the tris-buffered saline solution (Solarbio) with 0.1% (w/v) tween 20, an enhanced chemiluminescence detection kit (Beyotime) was used to visualize the protein blots using a multi-fluorescence and chemiluminescence imaging system (G: BOX Chemi XX6, Syngene). The resultant blots were quantified with Fiji.

DNA Fragmentation Detection

DNA fragments were detected by the agarose gel electrophoresis. Firstly, RM-1 cells were seeded in 6-well plates (3×10^5 cells per well) and pre-incubated for 12 h, followed by another 12 h of incubation in normoxia or hypoxia. Then the RM-1 cells were co-incubated with TPZ in various states for 12 h. After that, the DNA in washed cells was extracted using a DNA extraction kit (CWBio, China). The electrophoresis was performed on a gel composed of agarose at a concentration of 1.5% (w/v) in the Tris-borate-EDTA buffer solution. Lastly, the images were acquired with a multi-fluorescence imaging system.

Harvest of Animal Samples

Upon completion of therapy, the mice were anesthetized, and their peripheral blood was collected by picking off eyeballs. Following continuous cardiac perfusion sequentially using 20

mL PBS and 50 mL pre-cooled PFA solution, the major organs (heart, liver, spleen, lung, and kidney) and tumors of mice were sampled and immersed in the PFA solution. All the mice were sacrificed by cervical dislocation. The tumor inhibition rate was calculated based on the tumor weight and the formula: Tumor inhibition rate = $(W_{tumor, control}-W_{tumor, x})/W_{tumor, control} \times 100\%$, where $W_{tumor, control}$ signifies the average tumor weight of control group, and $W_{tumor, x}$ signifies the average tumor weight of *x* group. To conduct transcriptome sequencing, the sampled tumors without fixation were immediately washed with pre-cooled PBS, cut into pieces on ice, and quickly frozen in liquid nitrogen. Then, all mice were sacrificed by cervical dislocation. After fixation overnight at room temperature, spleens and tumors were gently washed with PBS and blotted dry with filter paper for the photographs and weighing.

Histology Evaluation

The fixed tissues and organs were shifted from the PFA solution into melted paraffin for embedding. The paraffin sections with a thickness of 7 μm were obtained using a Leica slicer (RM 2235, Germany). Following deparaffination and rehydration, the section slices were stained with an H&E staining kit (Servicebio, China). The TUNEL staining was performed with a TUNEL cell apoptosis detection kit (Servicebio). Before immunofluorescence staining, the rehydrated and antigen-repaired slices were blocked with normal goat serum for 30 min at room temperature. After washing, the slices were incubated with rabbit-derived primary antibodies against Ki67 (GB111141, Servicebio), HIF1α (AF7087, Beyotime), CD31 (GB11063-1, Servicebio), and MMP9 (AF5234, Beyotime) overnight at 4 °C. Subsequently, the washed slices were incubated with the goat-derived secondary antibody (anti-rabbit IgG) labeled with Alexa Fluor® 488 (GB25303, Servicebio) or Cyanine 3 (GB21303, Servicebio), followed by another washing. The washed slices were then mounted with an antifade solution with DAPI (Beyotime). At last, the fluorescence images of stained slices were captured with a fluorescence microscope (Ti2-U, Nikon) and quantified with Fiji.

Biosafety Evaluation

The PFA-fixed and paraffin-embedded organs were sectioned and stained with H&E. The sampled blood was stratified for 3 h at room temperature and centrifugated (1000 g) for 25 min at 4 °C. Following the collection of supernatants, the levels of ALT, AST, ALB, ALP, urea, CREA, CKMB, and LDH in the serums from mice undergoing different treatments were detected with an automatic biochemical analyzer (3100, Hitachi, Japan). The obtained index

level values were compared to the maximum serum biochemical indices suggested by the earlier studies.

Supplementary Figures

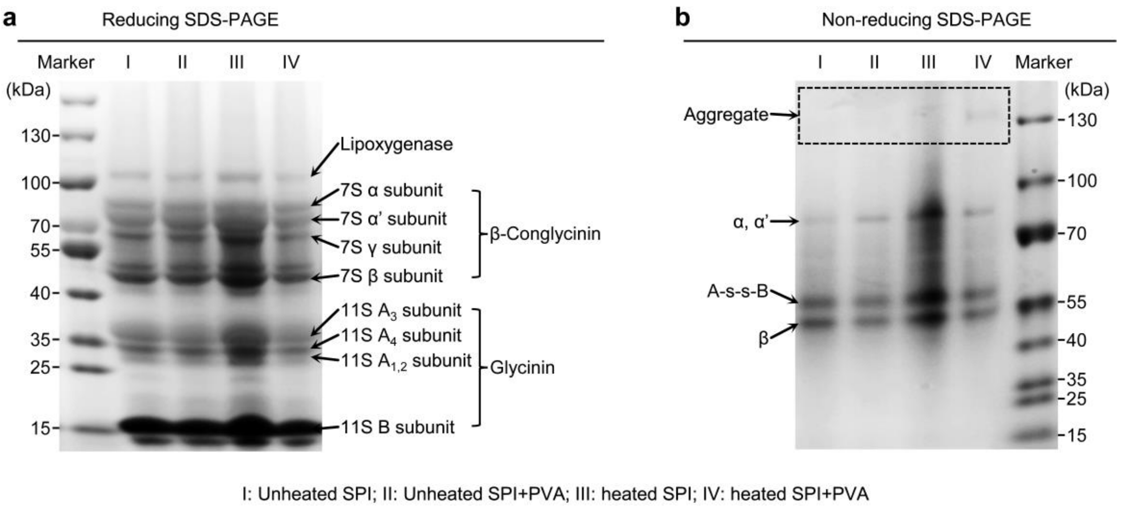

Figure S1. SDS-PAGE profiles of the individual SPI and the mix of SPI and PVA (w/w = 1:1). a) Reducing SDS-PAGE. b) Non-reducing SDS-PAGE.

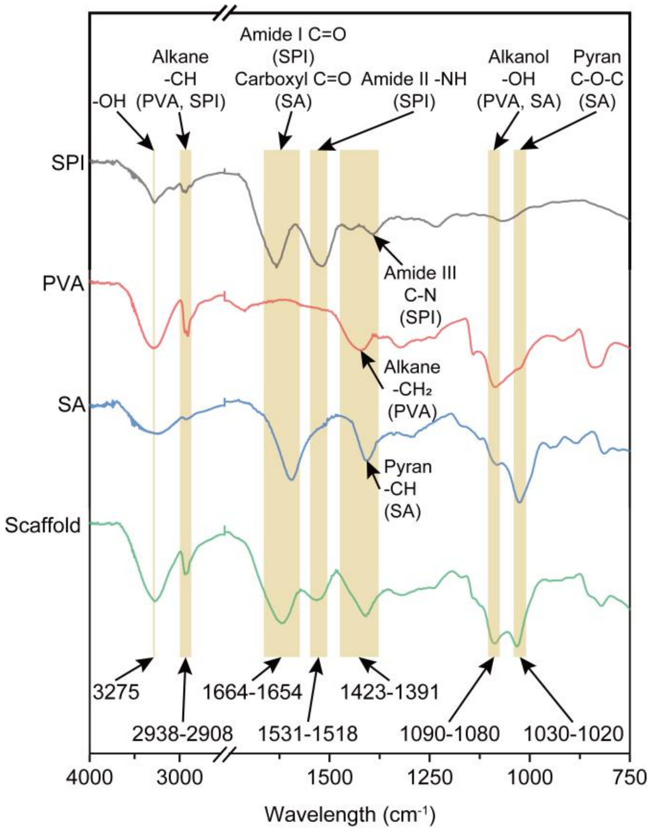

Figure S2. Fourier transform infrared (FTIR) spectra of SPI, PVA, SA, and the 3D-printed scaffold containing the three substances.

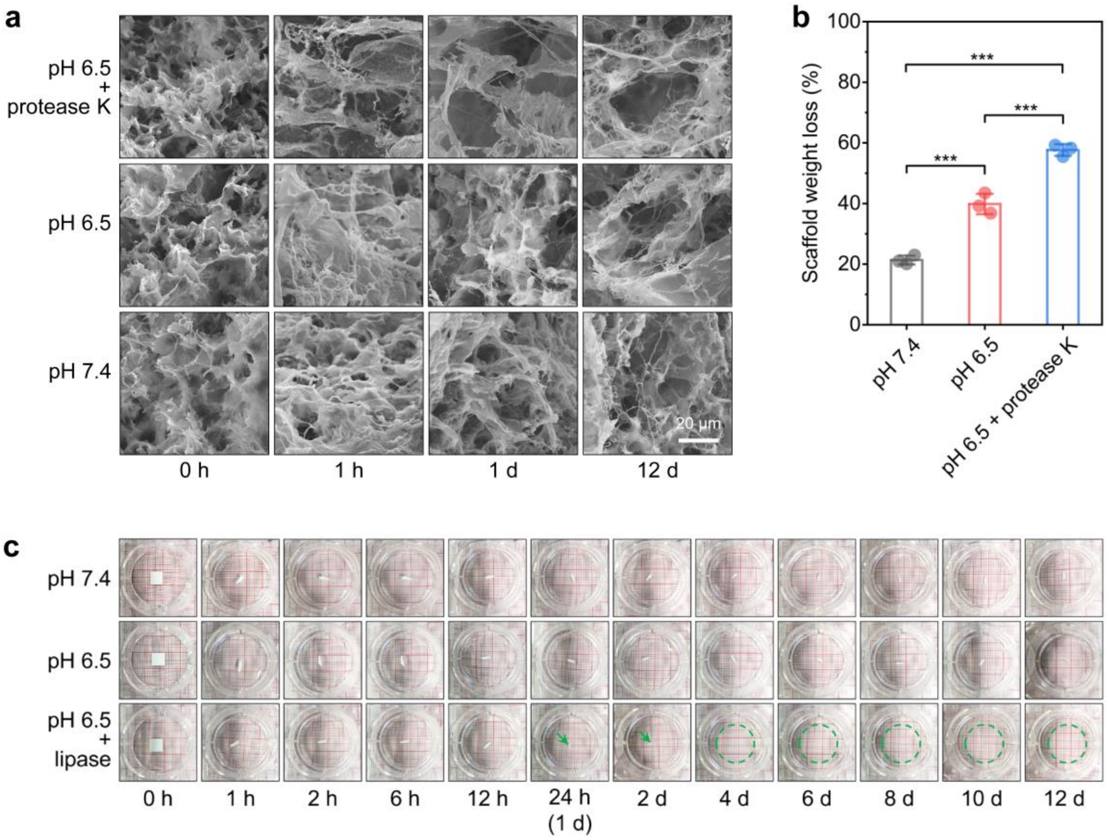

Figure S3. Degradability of scaffolds or fibers in vitro. Incubation time-dependent a) microtopography indicated by the SEM images of scaffolds in different PBS-based degradation liquids. b) Weight losses of scaffolds after incubation for 12 d in different PBS-based degradation liquids. c) Visual appearance over time for fibers in different PBS-based degradation liquids. PBS at pH 7.4 and 37 ^{o}C mimics the normal tissue microenvironment; PBS at pH 6.5 and 37 ^{o}C with or without protease K or lipase simulates tumor microenvironment. Small red grid references beneath the plates indicate 1 mm. Data are presented as mean ± SD (n = 3). Statistical significance was estimated by one-way ANOVA in the panel. ***$P < 0.001$.

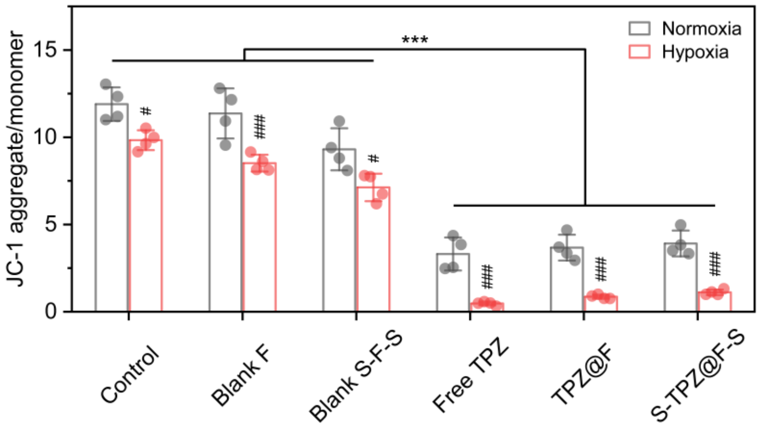

Figure S4. JC-1 aggregate/monomer ratios of mean fluorescence intensities for the RM-1 cells subjected to different treatments for 12 h at 37 °C, calculated based on the flow cytometry results. Data are presented as mean ± SD (n = 4). Statistical significance was estimated by two-way ANOVA. ***P < 0.001. #P < 0.05 and ###P < 0.001, by the intragroup comparison.

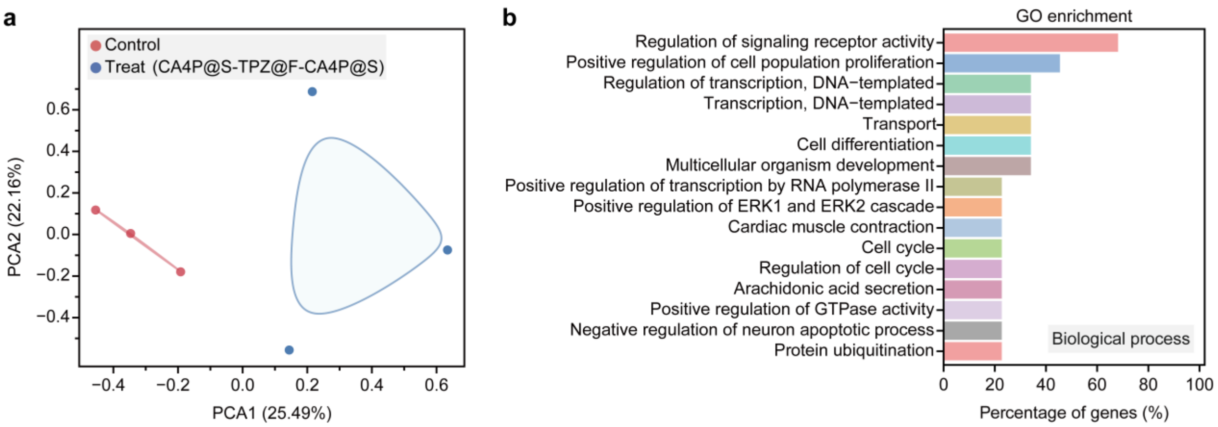

Figure S5. a) Principal component analysis (PCA) and b) GO term enrichment in the biological process of DEGs among the tumor samples from the control and CA4P@S-TPZ@F-CA4P@S groups (*n*=3 per group).

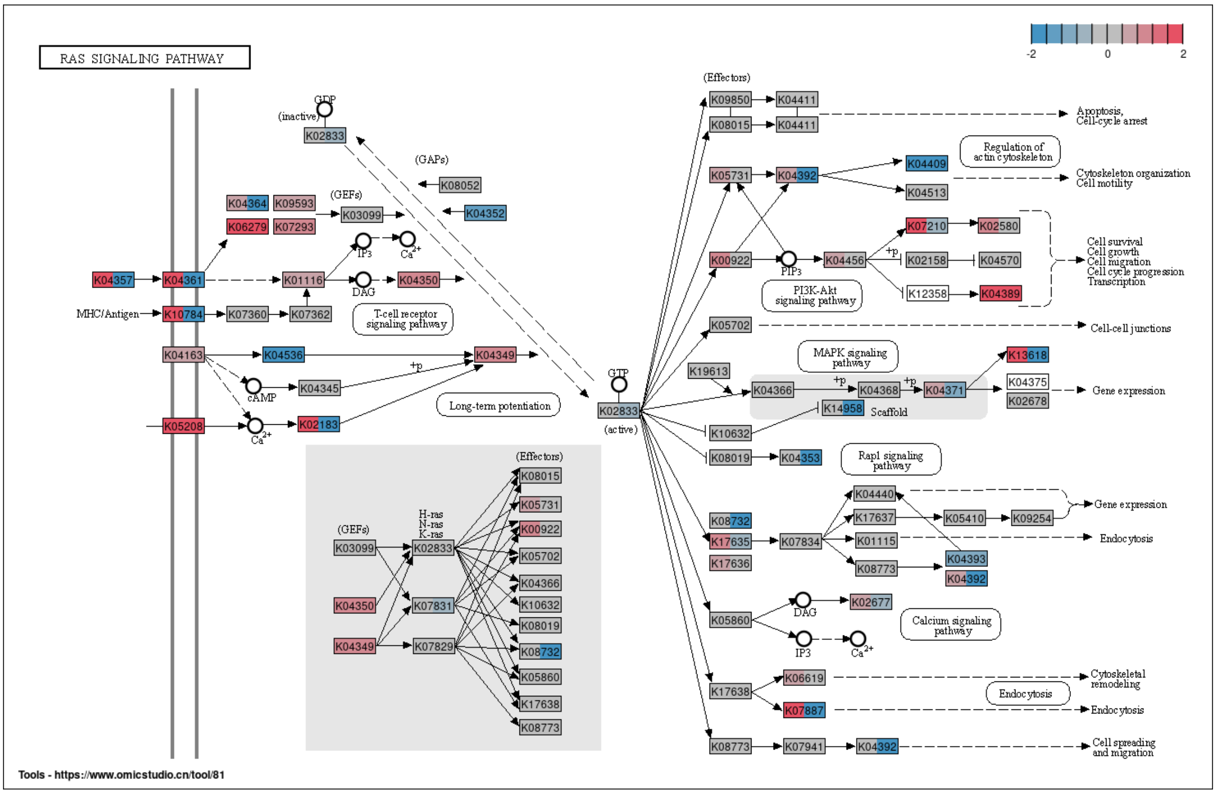

Figure S6. KEGG pathway of enriched Ras signaling pathway based on the DEGs among samples from control and CA4P@S-TPZ@F-CA4P@S groups.

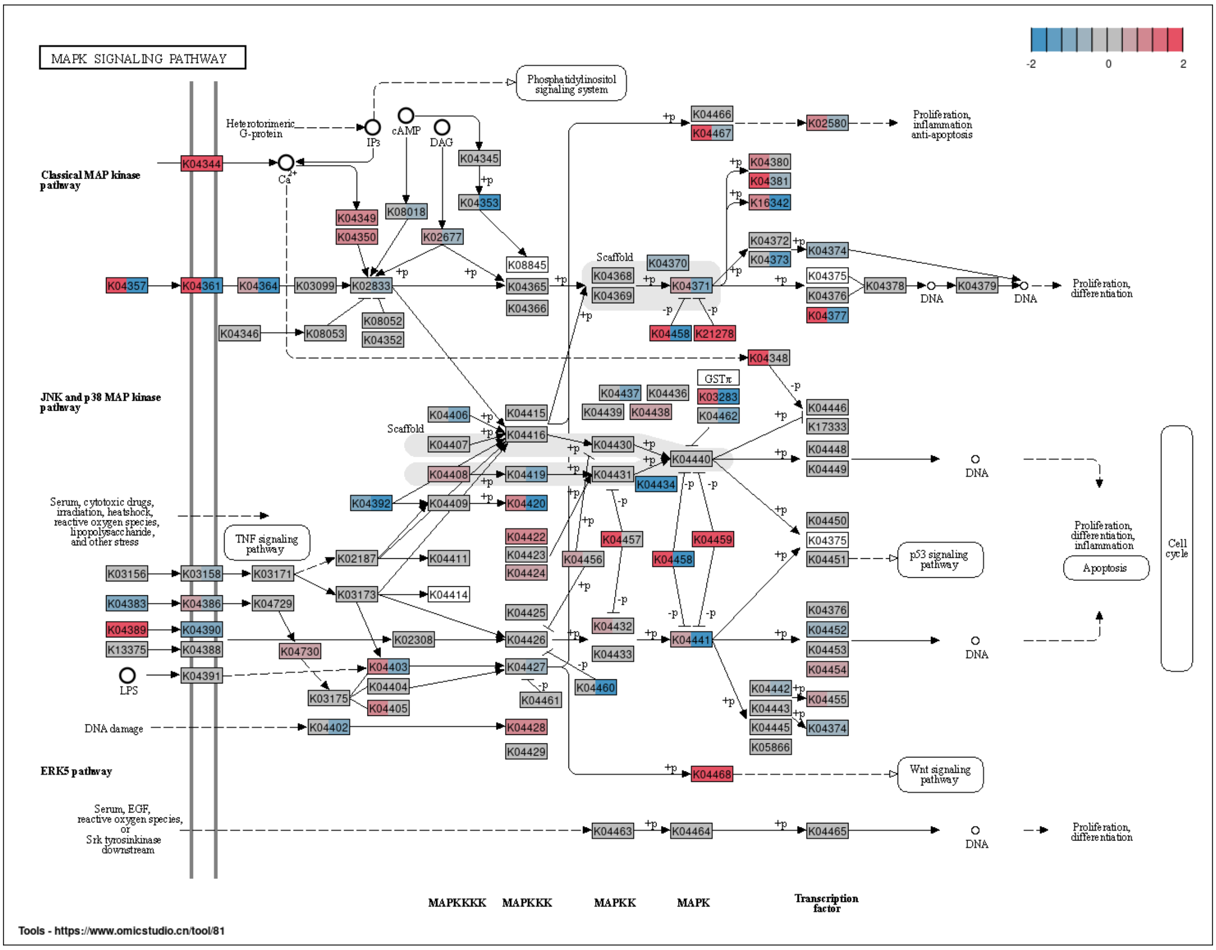

Figure S7. KEGG pathway of enriched MAPK signaling pathway based on the DEGs among samples from control and CA4P@S-TPZ@F-CA4P@S groups.

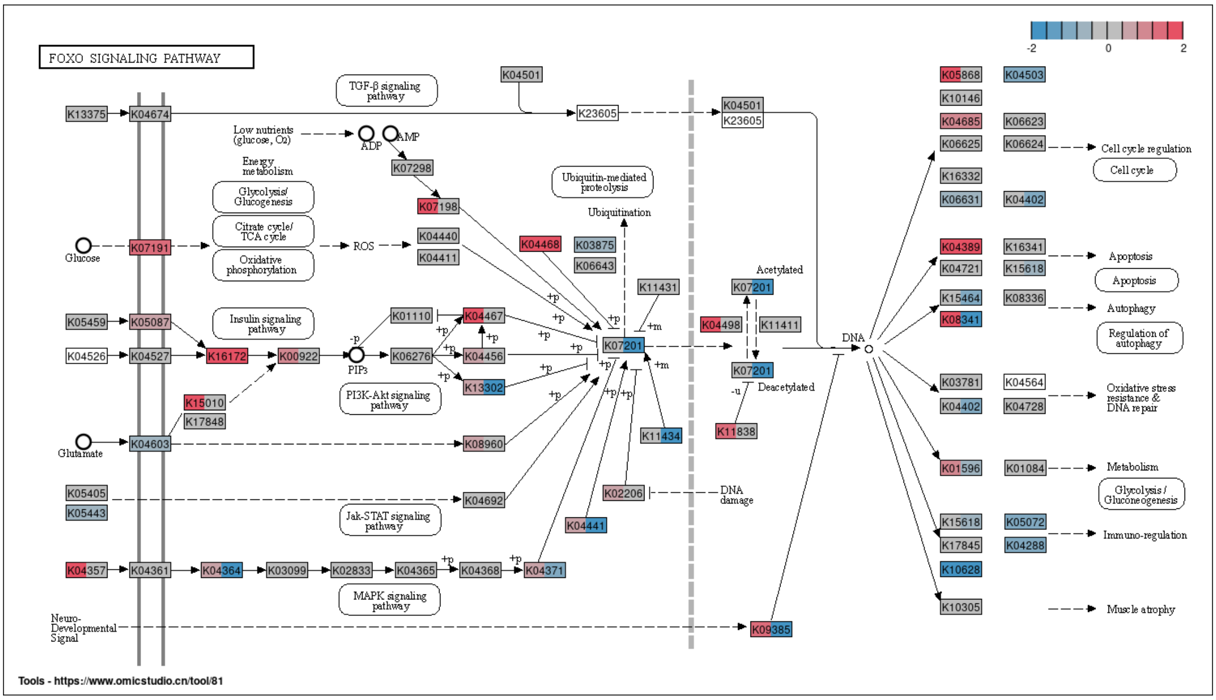

Figure S8. KEGG pathway of enriched FoxO signaling pathway based on the DEGs among samples from control and CA4P@S-TPZ@F-CA4P@S groups.

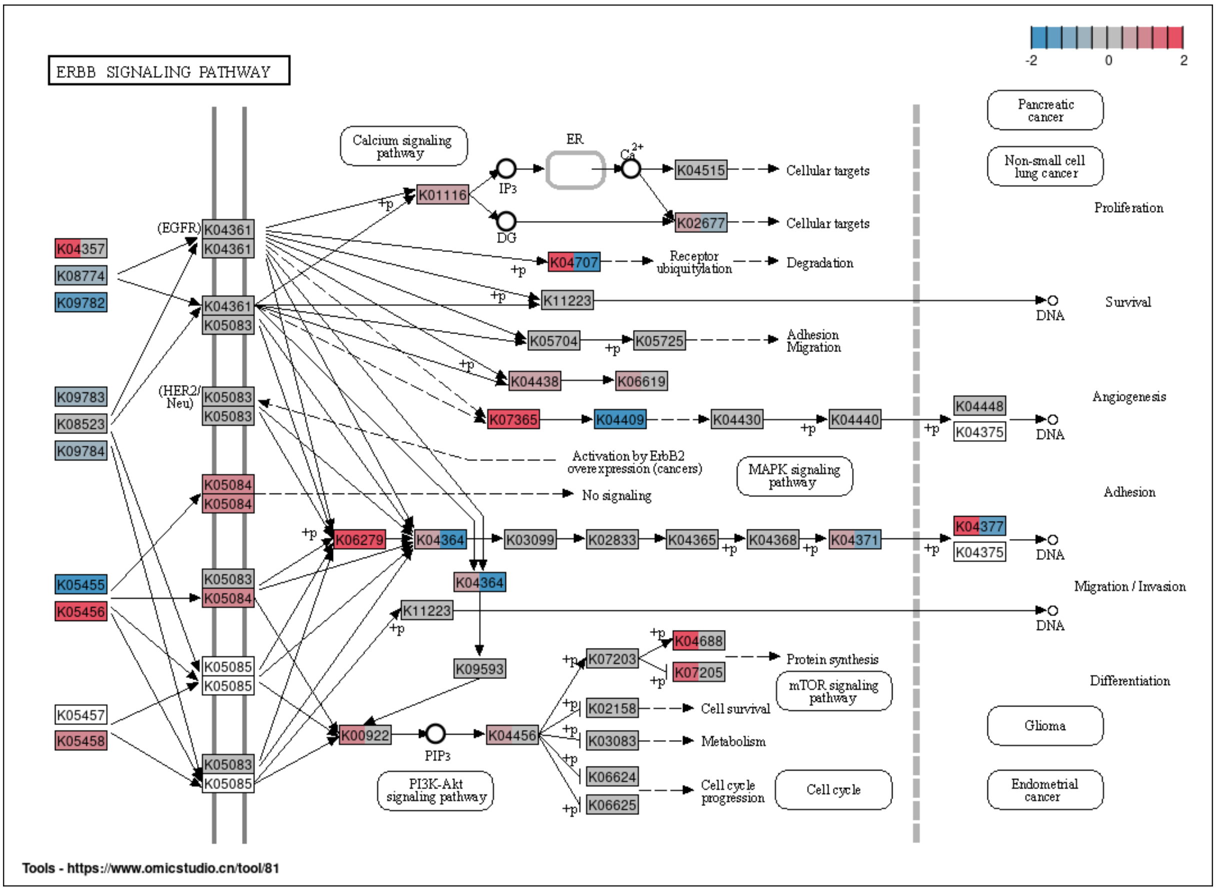

Figure S9. KEGG pathway of enriched ErbB signaling pathway based on the DEGs among samples from control and CA4P@S-TPZ@F-CA4P@S groups.

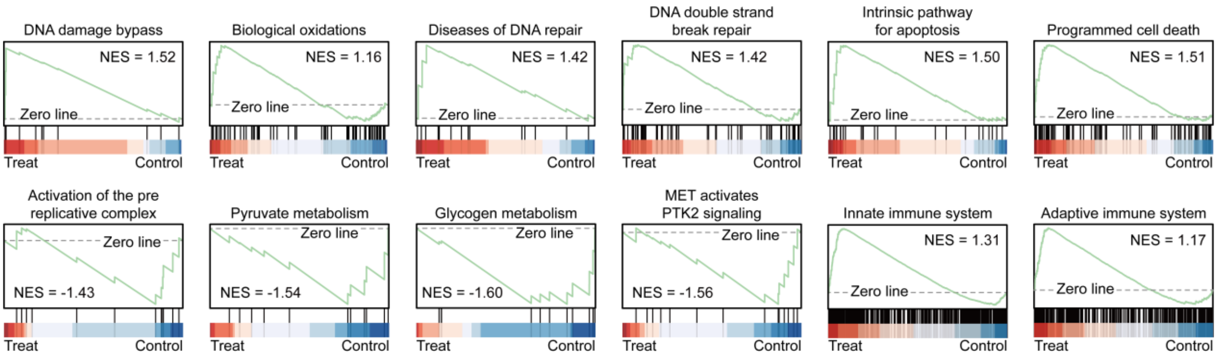

Figure S10. GSEA based on the Reactome pathway enrichment analysis showing upregulated or downregulated pathways [absolute value of normalized enrichment score (|NES|) > 1] related to potential tumor treatment mechanisms of CA4P or TPZ in the sandwich-structured implants.

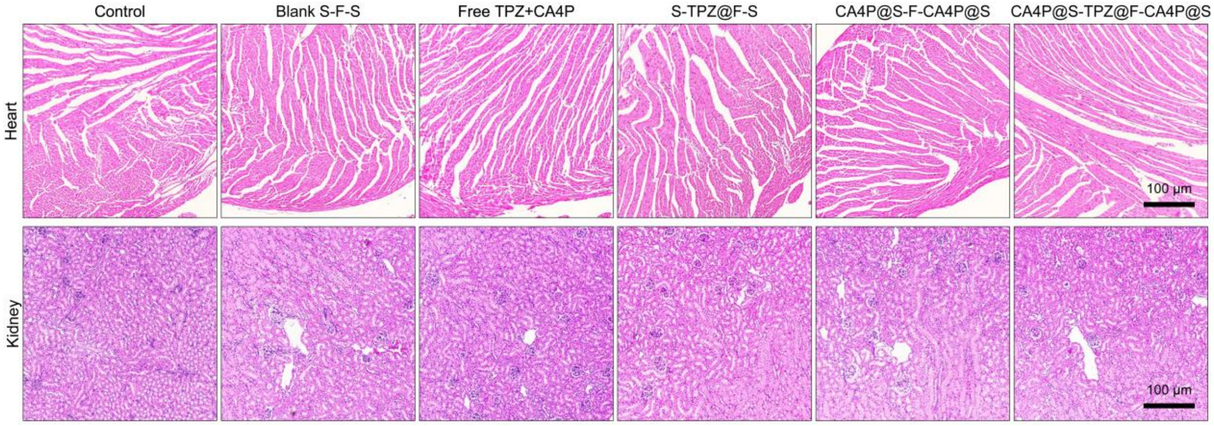

Figure S11. Representative images of H&E-stained tissue sections of hearts and kidneys from mice undergoing different treatments for 10 d, reflecting the biosafety via histological assessment.

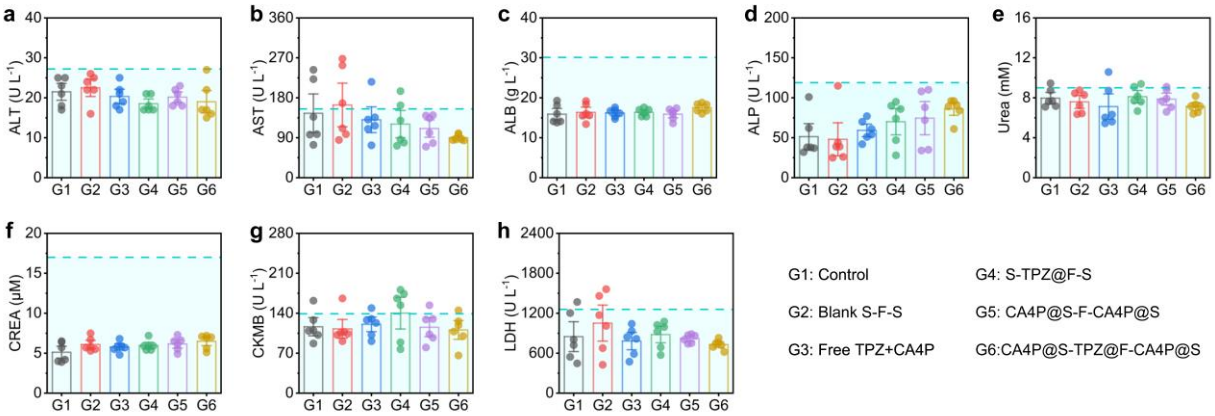

Figure S12. Levels of a) ALT, b) AST, c) ALB, d) ALP, e) urea, f) CREA, g) CKMB, and h) LDH in the serums from mice subjected to different therapies, demonstrating normal liver function (ALT, AST, ALB, and ALP), kidney function (urea and CREA), and heart function (CKMB and LDH) by serologic evaluation. Dash lines indicate the maximum levels of serum biochemical indices recommended by previous reports. The obtained index level values were compared to the maximum serum biochemical indices suggested by the earlier studies.[3] Data are presented as mean ± SD (n = 6).

References

[1] F. Liu, J. Zhu, P. Dai, J. Deng, J. Qin, Z. Yuchi, A. Fan, Z. Wang, Y. Zhao, *Adv. Funct. Mater.* **2021**, *31*, 2009157.

[2] a) D. B. Zorov, M. Juhaszova, S. J. Sollott, *Physiol. Rev.* **2014**, *94*, 909; b) D. C. Wallace, *Nat. Rev. Cancer* **2012**, *12*, 685.

[3] a) O. Boehm, B. Zur, A. Koch, N. Tran, R. Freyenhagen, M. Hartmann, K. Zacharowski, *Biol. Chem.* **2007**, *388*, 547; b) X. H. Zhou, G. K. Hansson, *Comp. Med.* **2004**, *54*, 176; c) E. Yazar, M. Elmas, V. Altunok, A. Sivrikaya, E. Oztekin, Y. O. Birdane, *Can. J. Vet. Res.* **2003**, *67*, 239; d) K. Schneck, M. Washington, D. Holder, K. Lodge, S. Motzel, *Comp. Med.* **2000**, *50*, 32.
